# Assessment of rhythmic entrainment at multiple timescales in dyslexia: Evidence for disruption to syllable timing^☆^

**DOI:** 10.1016/j.heares.2013.07.015

**Published:** 2014-02

**Authors:** Victoria Leong, Usha Goswami

**Affiliations:** Centre for Neuroscience in Education, Department of Psychology, University of Cambridge, Downing Street, Cambridge CB2 3EB, UK

## Abstract

Developmental dyslexia is associated with rhythmic difficulties, including impaired perception of beat patterns in music and prosodic stress patterns in speech. Spoken prosodic rhythm is cued by slow (<10 Hz) fluctuations in speech signal amplitude. Impaired neural oscillatory tracking of these slow amplitude modulation (AM) patterns is one plausible source of impaired rhythm tracking in dyslexia. Here, we characterise the temporal profile of the dyslexic rhythm deficit by examining rhythmic entrainment at multiple speech timescales. Adult dyslexic participants completed two experiments aimed at testing the perception and production of speech rhythm. In the perception task, participants tapped along to the beat of 4 metrically-regular nursery rhyme sentences. In the production task, participants produced the same 4 sentences in time to a metronome beat. Rhythmic entrainment was assessed using both traditional rhythmic indices and a novel AM-based measure, which utilised 3 dominant AM timescales in the speech signal each associated with a different phonological grain-sized unit (0.9–2.5 Hz, prosodic stress; 2.5–12 Hz, syllables; 12–40 Hz, phonemes). The AM-based measure revealed atypical rhythmic entrainment by dyslexic participants to *syllable* patterns in speech, in perception and production. In the perception task, both groups showed equally strong phase-locking to Syllable AM patterns, but dyslexic responses were entrained to a significantly *earlier* oscillatory phase angle than controls. In the production task, dyslexic utterances showed shorter syllable intervals, and differences in Syllable:Phoneme AM cross-frequency synchronisation. Our data support the view that rhythmic entrainment at slow (∼5 Hz, Syllable) rates is atypical in dyslexia, suggesting that neural mechanisms for syllable perception and production may also be atypical. These syllable timing deficits could contribute to the atypical development of phonological representations for spoken words, the central cognitive characteristic of developmental dyslexia across languages.

*This article is part of a Special Issue entitled <Music: A window into the hearing brain>.*

## Highlights

• Rhythmic entrainment at the syllable timescale is disrupted in dyslexia.
• Both syllable perception and production are atypical.
• Syllable timing deficits could contribute to dyslexics' atypical phonology.
• New AM-based methodology for measuring rhythmic entrainment is introduced.

## 1 Introduction

### 1.1 Hierarchical rhythm patterns in speech & music

There are myriad ways of defining rhythm, but for speech a frequently-utilised definition refers to an alternating pattern of ‘Strong’ and ‘weak’ elements (Schane, 1979; Lerdahl and Jackendoff, 1983). In the linguistic context, this rhythmic patterning occurs between successive syllables, which can be stressed (strong, ‘s’) or unstressed (weak, ‘w’). For example, the word “mi-ssi-ssi-ppi” contains 4 syllables that follow a ‘s–w–s–w’ pattern. This principle of strong–weak alternation also applies at higher levels of prosodic organisation, such as between successive prosodic ‘stress feet’ (motifs of strong and weak syllables). For example, the 4 syllables in “mi-ssi-ssi-ppi” may be grouped into 2 trochaic (‘s–w’) stress feet, where the 2nd stress foot (“ssi-ppi”) is more prominent than the 1st (“mi-ssi”). This results in a hierarchically-nested pattern such as ‘(s–w) (S–W)’, with brackets indicating stress feet and capital letters indicating greater relative prominence. In metrical phonology, these hierarchically-nested strong–weak prosodic patterns are typically represented as grids or trees (Selkirk, 1980, 1984, 1986; Liberman and Prince, 1977; Hayes, 1995). These hierarchical representations enable an analogy with metrical structure in music, which is also characterised by a strong–weak alternation of beats whose relative rhythmic prominence can also be expressed in hierarchical form (Lerdahl and Jackendoff, 1983).

The repetition of strong–weak beat patterns (for example in music or nursery rhymes) generates an acoustic framework of metrical regularity (Cooper and Meyer, 1960; Lerdahl and Jackendoff, 1983). Using this acoustic regularity structure, human activity (e.g. tapping or dancing) can become synchronised or ‘entrained’ to beat patterns in music and speech. The study of rhythmic entrainment and ‘sensorimotor synchronisation’ was pioneered in music research, typically using tapping tasks (see Repp, 2005 for a review). However, the concept of entrainment has recently gained wider significance in auditory neuroscience research as a basic mechanism for aligning endogenous neural oscillatory activity with salient events in the auditory environment, such as the acoustic cues to onsets of syllable units in speech (Lakatos et al., 2008; Schroeder and Lakatos, 2008, 2009; Luo and Poeppel, 2007; Zion Golumbic et al., 2012; Giraud and Poeppel, 2012). Here, we explore the relationship between rhythmic entrainment and the acquisition of written language (single word reading and spelling) in adults with and without developmental dyslexia.

### 1.2 Dyslexia and rhythm

Children with developmental dyslexia have difficulty in the accurate neural representation of phonological aspects of speech, across languages (see Ziegler and Goswami, 2005; for review). Phonological awareness, the ability to recognise and manipulate sound units in words, follows a developmental sequence in all languages so far studied, from syllable to onset-rime to (once reading is taught) phoneme. Therefore, disruption to syllable timing (arising, for example, from the inaccurate localisation of syllable boundaries) would have a cascading effect on spoken word representations over the time-course of development, leading to the atypical neural specification of phonology at every phonological level. Indeed, dyslexia is characterised by the inefficient development of the entire phonological system (encompassing the accurate specification of phonology in words, efficient phonological memory, and efficient and rapid output of automatized phonological information such as over-learned colour names, see Snowling, 2000). Recently, the phonological difficulties characterising individuals with dyslexia have begun to be studied in terms of sensitivity to speech prosody and rhythm (e.g. Wood and Terrell, 1998; Kitzen, 2001; Goswami et al., 2010, 2013a; Leong et al., 2011a; Holliman et al., 2010, 2012). These studies consistently indicate that individuals with reading difficulties (both children and adults) show reduced sensitivity to speech rhythm and prosody, specifically to strong–weak syllable stress patterns. Moreover, poor prosodic sensitivity is typically a strong predictor or correlate of poor reading and phonology.

Rhythm deficits in speech *production* have also been observed in dyslexia. For example, Smith et al. (2008) documented speech timing difficulties in 2- and 3-year-old children at family risk for dyslexia, who later presented with reading difficulties. At age 2 and 3 years, these children produced significantly fewer syllables per second than no-risk children and paused for longer between articulations, suggestive of early syllable-level deficits. Similarly, 3-year old Dutch children at risk of dyslexia also showed difficulties when asked to imitate non-words with irregular stress patterns (de Bree et al., 2006). Wolff (2002) asked adults with dyslexia to repeat motifs of stressed and un-stressed syllables, such as “PA pa pa” or “pa pa PA pa” (stressed syllable in capital letters). Wolff (2002) reported that dyslexic participants produced more errors in stress assignment, and showed significantly lower amplitude increments for stressed versus unstressed syllables. The rhythm deficit in dyslexia also extends to musical and non-speech tasks. For example, dyslexics were significantly more erratic than controls when asked to tap in time to an external pacing metronome (1.5 Hz, 2 Hz, 2.5 Hz), and individual differences in rhythmic entrainment were related to individual differences in reading development (Thomson et al., 2006; Thomson and Goswami, 2008). In two studies of musical metrical sensitivity, dyslexic children were significantly poorer than controls at detecting violations to musical metrical structure in tone sequences (Huss et al., 2011; Goswami et al., 2013b). Furthermore, metrical sensitivity predicted phonological awareness and reading in both studies, accounting for over 60% of variance in reading along with age and I.Q. for younger children (Huss et al., 2011).

The relationship between prosodic rhythm awareness and phonology may be rooted in events that shape the language system during infant development. Human speech heard from inside the womb is effectively low-pass filtered by the uterine wall, isolating low frequency information and accordingly foregrounding prosodic and rhythmic structure (Armitage et al., 1980). Therefore, even while in the womb, infants are already being exposed to the global prosodic patterns of their native language. Prosodic rhythm patterns in speech (particularly syllable stress patterns) are thought to play an important role in ‘bootstrapping’ early language acquisition (Gleitman and Wanner, 1982). It has been suggested that infants use the most representative prosodic stress patterns of their native language to parse the speech signal into candidate words via a ‘metrical segmentation strategy’ (Cutler and Norris, 1988). For example, in the English language, around 90% of content words begin with a strong initial stressed syllable, such as “DA-ddy” or “BA-by” (Cutler and Carter, 1987). By 9 months of age, English-learning infants show sensitivity to this prosodic statistic, preferring words with a trochaic ‘strong–weak’ (s–w) syllable pattern over those with an iambic ‘weak–strong’ (w–s) syllable pattern (Jusczyk et al., 1993; Echols et al., 1997). English learning infants also preferentially use the trochaic motif as a word segmentation strategy (Jusczyk et al., 1999). Therefore, infants with a reduced sensitivity to prosodic stress patterns in speech may be expected to struggle with a prosodic-based speech segmentation strategy. Over development, this could result in differently-specified mental representations of phonological aspects of speech (as observed in dyslexia).

### 1.3 Acoustic cues for speech rhythm perception

In terms of the acoustic cues to prosody and stress in speech, it is known that stressed syllables tend to be higher in amplitude, longer in duration and have wider excursions in fundamental frequency (Hirst, 2006). Therefore, the alternating ‘strong–weak’ syllable patterns that generate the percept of speech rhythm are obviously associated with patterns of change in all three acoustic dimensions (amplitude, duration and frequency). Traditionally, fundamental frequency was thought to play a primary role in prosodic stress perception (Fry, 1955, 1958). However, more recent studies using natural speech have found that amplitude and duration cues (which typically co-vary) play a stronger role than fundamental frequency in prosodic prominence, and by extension in speech rhythm (Greenberg, 1999; Kochanski et al., 2005). Accordingly, methods of describing and measuring speech rhythm can be broadly classified as being either duration-based or amplitude-based in approach. The duration-based approach is typified by ‘rhythm-metric’ measures. These are summary statistics designed to distinguish between languages with different perceived rhythmic qualities in terms of consonantal (C) and vocalic (V) duration, such as ‘stress-timed’ versus ‘syllable-timed’ languages (Abercrombie, 1967; Pike, 1945). For example, indices like %V, ΔV, ΔC (Ramus et al., 1999) quantify the relative proportions of vocalic intervals and the standard deviation of vocalic and consonantal durations in speech, while pairwise variability indices (PVIs, Grabe and Low, 2002) and rate-normalized measures like VarcoV and VarcoC (Dellwo and Wagner, 2003) focus on the relative variability in the length of successive consonantal and vocalic intervals.

By contrast, amplitude-based approaches have traditionally been used in the measurement of perceptual centres or ‘P-centres’. The P-centre refers to the perceptual ‘moment of occurrence’ of events in any sensory modality (Morton et al., 1976; Marcus, 1981), and is thus closely associated with beat perception in music and speech. The P-centre or beat of a sound is not perceived at the exact instant that the sound begins to be produced. Rather, the P-centre is perceived a short time after the sound onset, before the sound reaches its maximum loudness (amplitude). That is, the P-centre is located at some point along the initial slope of rising amplitude, or 'rise time slope' (Scott, 1993). In speech, syllable P-centres are typically located *near* the onsets of vowel nuclei (Allen, 1972; Marcus, 1981; Port, 2003), though the exact location may be influenced by preceding and following consonants.

With respect to dyslexia, it has been suggested that sensory insensitivity to amplitude rise time changes in acoustic signals could be related to prosodic and rhythmic deficits (Goswami et al., 2002; Goswami, 2011). In the context of speech, impaired sensitivity to amplitude modulation (AM) patterns would be expected to impair dyslexics' detection of syllable P-centres, affecting performance in syllable counting or tapping tasks, as well as dyslexics' ability to use amplitude-based cues to distinguish between stressed and unstressed syllables. Consistent with this view, the detection of auditory rise time changes has been found to be impaired in dyslexic individuals, across languages as diverse as English, Spanish, Chinese, Finnish, French, Hungarian and Dutch (Goswami et al., 2002, 2011; Goswami, 2011; Muneaux et al., 2004; Surányi et al., 2009; Poelmans et al., 2011; Hämäläinen et al., 2012a). Moreover, in English, individual differences in rise time sensitivity predict dyslexics' performance in rhythm-based tasks (Goswami et al., 2010; Leong et al., 2011a; Huss et al., 2011). The close association between amplitude changes in the acoustic signal and rhythmic beat (P-centre) perception has also prompted amplitude-based approaches to characterising rhythm and prosodic patterning in speech (Silipo and Greenberg, 1999; Tilsen and Johnson, 2008; Leong, 2012).

### 1.4 Measuring rhythm from the speech amplitude envelope

In signal processing terms, the speech signal can be modelled as the product of a quickly-varying carrier (fine structure) and a more slowly-varying amplitude envelope that dynamically modulates the amplitude of the carrier. This envelope-carrier decomposition (termed ‘demodulation’) provides a convenient way to isolate the amplitude-based cues to syllable P-centres and prosodic rhythm patterns found in the original speech signal. The envelope itself contains multiple rates of amplitude modulation (AM), forming a ‘modulation spectrum’ of different modulation rates, not all of which are equally important for transmitting rhythm. In this modulation spectrum, the strongest modulation is typically observed at around 3–5 Hz irrespective of differences in language or speech rate (Shannon et al., 1995; Houtgast and Steeneken, 1985; Greenberg et al., 2003; Greenberg, 2006). As the average duration of a syllable is 200 ms, these amplitude modulations around 5 Hz are likely to relate to syllable-pattern information in speech (Greenberg et al., 2003; Ahissar et al., 2001; Luo and Poeppel, 2007). Amplitude modulations slower than the syllable rate are thought to relate to prosodic stress patterns (Greenberg et al., 2003; Ghitza and Greenberg, 2009) whereas faster AMs up to 50 Hz contain linguistic cues to phonetic manner of articulation, voicing, and vowel identity (Rosen, 1992). The role of different AM rates within the speech envelope is usually investigated with regard to speech intelligibility. For example, in two seminal studies, Drullman and colleagues (Drullman et al., 1994a, 1994b) used systematic low- and high-pass filtering of the amplitude envelope to define the range of modulation rates that are the most important for good speech intelligibility (∼4–16 Hz). Other studies (e.g. Shannon et al., 1995) have used ‘vocoding’ approaches (combining the demodulated envelope from different acoustic frequency bands with noise or pure tones) to investigate the minimum number of acoustic channels that are required for good phonetic discrimination (∼3 channels).

Recently, interest has grown in the recovery of *rhythm* cues (rather than intelligibility cues per se) from the speech amplitude envelope (Tilsen and Johnson, 2008; Leong et al., 2011b; Leong, 2012). These envelope-based rhythm cues help to support rhythmic synchronisation between speakers even when speech is unintelligible (Cummins, 2009). One such novel approach, the Spectral Amplitude Modulation Phase Hierarchy (S-AMPH) model (Leong, 2012) is used here (described in Appendix 1). In the S-AMPH model, an AM hierarchy consisting of nested modulation patterns at ‘Stress’ (0.9–2.5 Hz), ‘Syllable’ (2.5–12 Hz) and ‘Phoneme’ (12–40 Hz) rates is extracted from the speech amplitude envelope. These 3 AM tiers represent the dominant non-redundant modulation structure that is present in the speech envelope at 3 different (but concurrent) timescales. Leong (2012) determined the existence of these AM tiers via principal component analysis of a multi-speaker corpus. By symmetry to the linguistic prosodic hierarchy, each AM tier corresponds well to the characteristic timescale of a different-sized phonological unit: the prosodic stress foot (∼2 Hz, Dauer, 1983), syllable (∼5 Hz, Greenberg et al., 2003) and phoneme (e.g. consonants ∼14 Hz, Crystal and House, 1988). The oscillatory patterns of the slower ‘Stress’ and ‘Syllable’ AM tiers can then be used to infer rhythm-related speech activity. For example, Leong (2012) found that oscillatory peaks within the ‘Syllable’ AM can be used to assess the location of individual syllable vowel nuclei, with 82–98% accuracy (for freely-produced and metronome-timed speech respectively). Also, instantaneous oscillatory *phase* relationships between the ‘Stress’ AM and the ‘Syllable’ AM can be used to infer ‘strong–weak’ syllable stress patterns, with 70–94% accuracy. Here we use the novel S-AMPH method to assess entrainment to speech rhythm patterns on multiple timescales. Neurally, these dynamic AM patterns in the amplitude envelope could also be important for entraining endogenous neuronal oscillatory activity, which would align brain activity with on-going prosodic, syllabic and phonetic patterns in speech (Poeppel, 2003; Giraud and Poeppel, 2012).

### 1.5 Multi-timescale neuronal oscillatory entrainment to speech

Speech contains important temporal structure at different timescales, as exemplified by phonemes (timescale tens of ms), syllables (timescale hundreds of ms) and prosodic stress patterns (timescale seconds). A recent neural model of speech perception has proposed that the brain performs temporal sampling of the speech signal at *multiple time-scales*, to simultaneously capture these phonological elements of different grain size (Poeppel, 2003; Giraud and Poeppel, 2012). According to this theory, temporal sampling is effected by the endogenous oscillatory activity in the auditory cortex, which ‘entrains’ (via phase-locking) to the spectro-temporal modulation patterns associated with different phonological grain-sized elements in speech. This neuronal oscillatory activity arises from fluctuations in the local field potential of neuronal populations, and is observed to be concentrated within certain characteristic frequency bands (Buzsaki and Draguhn, 2004). Of particular salience to the theory are the neural oscillatory ‘delta’ (1–3 Hz), ‘theta’ (4–7 Hz) and ‘gamma’ bands (25–80 Hz), which have been implicated in the temporal sampling of prosodic, syllabic and phonemic speech information respectively (Giraud and Poeppel, 2012; Ghitza and Greenberg, 2009; Ghitza, 2011). Consistent with this theory, the strength of theta band (syllable-rate) phase-locking has been associated with speech intelligibility in several human MEG studies (Ahissar et al., 2001; Luo and Poeppel, 2007; Luo et al., 2010). Moreover, auditory stimuli may be processed differently depending on the phase of underlying neuronal oscillatory activity. For example, Henry and Obleser (2012) found that individual listeners' ability to detect gaps in a frequency-modulated sound was dependent on their instantaneous phase of entrained delta oscillations in the cortex.

Furthermore, in primate studies, auditory cortical oscillatory activity in delta, theta and gamma bands has been observed to show *hierarchical-nesting* (Lakatos et al., 2005; Kayser et al., 2009), such that the oscillatory phase of a slower band (e.g. theta) dynamically modulates the oscillatory power of a faster band (e.g. gamma). This phase-nesting between oscillatory rates is thought to stabilise auditory sensory representations (Kayser et al., 2009), and to facilitate multi-timescale integration and synchronisation of the acoustic information sampled at different rates, both within the auditory domain and in auditory-visual speech integration (Lakatos et al., 2005; Palva et al., 2005; Canolty and Knight, 2010). However, the role of cross-frequency neuronal synchronisation/hierarchical nesting in human speech perception has not been much tested (Luo et al., 2010).

Recently, it has been proposed that the phonological deficits found in developmental dyslexia across languages may arise in part because of atypical ‘temporal sampling’ of the speech signal by neuronal oscillations, specifically at slower syllable- (theta) and stress-related (delta) rates below 10 Hz (Goswami, 2011). Following AM accounts (e.g. Ghitza and Greenberg, 2009), this impaired neural sampling should affect the efficient recovery of *syllabic and prosodic structure* from the speech signal, consistent with previous behavioural studies indicating reduced sensitivity to syllable stress patterns in dyslexia (Wood and Terrell, 1998; Kitzen, 2001; Goswami et al., 2010, 2013a; Leong et al., 2011a; Holliman et al., 2010, 2012). In support of Goswami's proposal, individuals with dyslexia show significantly reduced phase locking within the delta range (2 Hz) in response to amplitude-modulated white noise (Hämäläinen et al., 2012b). However, reduced gamma-rate sampling has also been proposed to be a causal factor in dyslexia (Lehongre et al., 2011). Given the hierarchical nesting between slow (theta) and fast (gamma) oscillatory rates in the cortex, abnormalities in temporal sampling at the theta rate may invariably result in altered gamma-rate activity.

### 1.6 Multi-timescale rhythmic entrainment in dyslexia

Here, we investigate the behavioural hallmarks of multi-timescale rhythmic entrainment in dyslexia, exploring both speech rhythm perception (Experiment 1) and speech rhythm production (Experiment 2). If atypical neuronal oscillatory entrainment at slow rates (<10 Hz) relates to the observed rhythm deficit in dyslexia, we should observe an altered entrainment phenotype. Entrainment should be atypical primarily at slower ‘Stress’ and ‘Syllable’ timescales, rather than at the faster ‘Phoneme’ timescale (Goswami, 2011). Furthermore, given the hierarchically-nested nature of neuronal oscillations (Lakatos et al., 2005), disruptions to slower neuronal oscillations in dyslexia should produce atypical acoustic cross-frequency phase-locking between slow (e.g. theta – syllable rate) and fast (e.g. gamma – phoneme rate) oscillations. This altered neuronal cross-frequency nesting may result in observable effects in dyslexics' produced speech, since speech perception and production mechanisms are thought to share similar rhythmic constraints and neural representations (Martin, 1972; Liberman and Mattingly, 1985). An underlying difference in entrainment to slow rhythms could be expressed for example as atypical temporal synchronisation of phonemic units within the syllable unit, or atypical temporal synchronisation of syllable units within the stress foot.

To investigate these hypotheses, we tested adults with and without dyslexia in two speech rhythm experiments. In a rhythm perception experiment, participants were asked to tap along to the beat of 4 metrically-regular nursery rhyme sentences. In a rhythm production experiment, participants were asked to speak aloud the same 4 nursery rhyme sentences in time with a metronome beat. All participants performed the production experiment before the perception experiment, so that their utterances would be unbiased by the exemplars heard in the perception experiment. However, we present the results of the perception experiment first. Control and dyslexic responses were compared in each experiment using both traditional and multi-timescale (envelope AM-based) entrainment measures. Nursery rhyme sentences were used as experimental stimuli because they contain regular strong–weak syllable stress patterns, facilitating rhythmic entrainment in participants. In addition to the two rhythm experiments, participants also completed standardised tests for general ability (IQ), reading, spelling and phonological awareness.

## 2 Participant characteristics

21 adults with dyslexia (9 M, 12 F), and 22 adults without dyslexia (7 M, 15 F) participated. Dyslexic participants had a formal statement of developmental dyslexia, were native English speakers, and had no other documented learning disabilities or hearing difficulties (assessed by self-report). Control and dyslexic groups were matched for mean chronological age (controls ranged in age from 20.1 to 29.5 years, mean 24.1 years; dyslexics ranged in age from 19.6 to 29.7 years, mean 22.9 years). To ensure that our dyslexic participants did indeed have significant reading and phonological problems (relative to the control group), but otherwise showed normal cognitive ability, we administered a set of standardised tasks to all participants. These comprised two subscales of the Wechsler Abbreviated Scale of Intelligence (WASI; Wechsler, 1999: *Block Design* [Control standardised T-score = 70.59, s.d. 4.14; Dyslexics = 70.57, s.d. 3.03] and *Vocabulary* [Control standardised T-score = 62.09, s.d. 7.86; Dyslexics = 62.04, s.d. 4.71]), the untimed Wide Range Achievement Test (single word Reading and Spelling scales, WRAT-III, Wilkinson, 1993; Control reading standard score = 115.59, s.d. 5.34; Dyslexics = 110.81, s.d. 6.44, Control spelling standard score = 116.45, s.d. 6.07; Dyslexics = 104.71, s.d. 6.67), and a standardised phonological awareness measure (the *Spoonerism* task from the Phonological Assessment Battery, PhAB; Fredrickson et al., 1997; Control score out of 30 = 28.5, s.d. 1.41; Dyslexics = 26.1, s.d. 2.05). Group performance on the 3 standardised literacy & phonology tasks was compared using a MANOVA, which revealed a significant overall group difference (Wilks' *λ* = 0.44, *F* (3, 39) = 16.3, *p* < 0.001), Univariate results confirmed that our recruited dyslexic cohort did indeed suffer from significant reading (*F*[1,41] = 7.1, *p* < 0.05), spelling (*F*[1,41] = 36.5, *p* < 0.001) and phonological problems (*F*[1,41] = 20.3, *p* < 0.001), despite being of similar intelligence to the control group.

## 3 Experiment 1: speech rhythm perception (tapping)

### 3.1 Rationale

In this experiment, we aimed to elucidate differences between control and dyslexic participants in terms of *entrainment* (tapping) to speech rhythm patterns on different timescales. Accordingly, we presented participants with speech samples that possessed a strong rhythmic beat (nursery rhymes). We then asked participants to detect the spoken rhythm, and tap along to the beat that they perceived.

### 3.2 Methods

#### 3.2.1 Materials

**Fig. 1:**
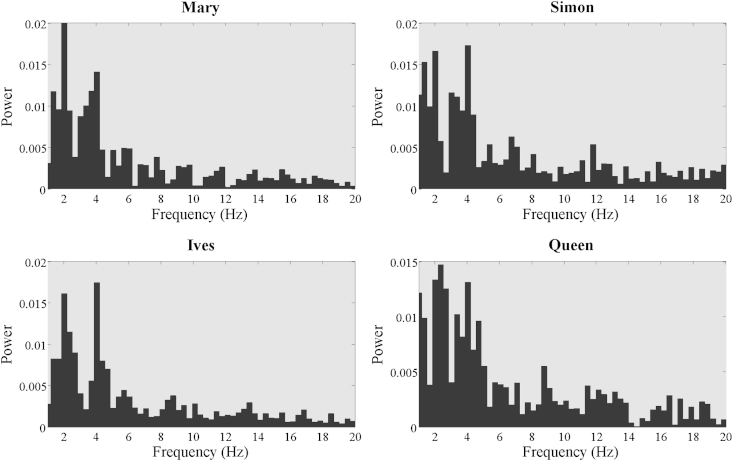
Computed envelope modulation spectrum for each of the 4 nursery rhyme sentences. All sentences show peaks in their spectra at around 2 Hz and 4 Hz, corresponding to the metronome-timed stress rate and syllable rate of the utterance respectively.

Four nursery rhyme sentences were used in this rhythm perception task. Sentences were 8 syllables in length and had a binary alternating rhythm of strong (s) and weak (w) syllables. Two sentences (“Mary Mary quite contrary” and “Simple Simon met a pieman”) had a trochaic stress pattern (‘s–w–s–w–s–w–s–w’) while the other two sentences (“as I was going to St Ives” and “the Queen of Hearts she made some tarts”) had an iambic stress pattern (‘w–s–w–s–w–s–w–s’). In this rhythm perception task, participants heard audio recordings of the four sentences. These were naturally produced by a female native British English speaker who was speaking in time to a 4 Hz (syllable rate) metronome beat. Therefore the four sentences were highly metrically-regular, with syllables occurring approximately every 250 ms (4 Hz), and stressed syllables occurring approximately every 500 ms (2 Hz). However, the metronome beat was not audible in the final recording. Each sentence had a duration of around 2 s. Fig. 1 shows the envelope modulation spectrum of the 4 nursery rhyme sentences, derived by computing the Fourier transform of the Hilbert envelope of each utterance. As expected, there are strong peaks in the power of the envelope for each sentence at around 2 Hz and 4 Hz, corresponding to the rate of stress and syllable patterning respectively.

#### 3.2.2 Task description

**Fig. 2:**
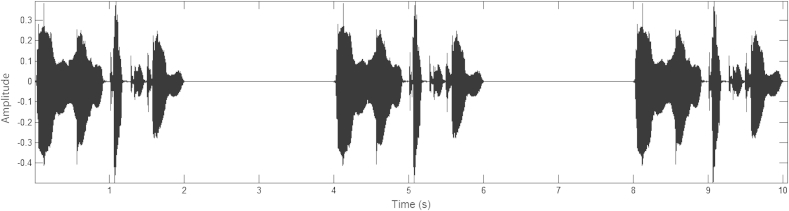
Example of the acoustic stimulus for a single tapping trial. The nursery rhyme sentence was ‘Mary Mary quite contrary’, repeated 3 times.

In this task, participants heard the nursery rhyme sentences, and were asked to tap along to the rhythm of each sentence. During a single trial, one nursery rhyme sentence was repeated three times, with a silent gap between repetitions that was equal to the length of that sentence (around 2 s). Participants were asked to maintain their tapping during these silent periods, and to aim to come back in on time with the next occurrence of the sentence. Fig. 2 shows an example of a trial for the sentence “*Mary Mary quite contrary*”, as presented to participants. Here, the length of the original sentence was 2.01 s, and this was also the length of silence inserted between repetitions of the sentence. Participants were instructed to begin tapping as soon as they heard the sentence begin. No instructions were given as to the rate of tapping, but all participants spontaneously tapped according to the *stress* rate of the sentence (i.e. 2 Hz), rather than the syllable rate (i.e. 4 Hz). It is important to note that the sentences used here did not contain an audible metronome beat, but were recordings of rhythmic speech produced to a metronome beat – that is, speech with a clear beat. Therefore, this task tested perception of and entrainment to the *acoustic carriers* of rhythmic beats in speech (e.g. AM patterns).

The two trochaic sentences were presented first before the two iambic sentences, as the trochaic sentences were easier to track rhythmically. However, the order of presentation within the pairs of trochaic and iambic sentences (i.e. ‘Mary Mary’ first or ‘Simple Simon’ first) was counterbalanced across participants. Therefore, each participant completed 4 trials in total, each containing 3 repetitions of a single nursery rhyme sentence. The stimuli were presented binaurally using Sennheiser HD580 headphones at 70 db SPL. The task was programmed using Presentation software (Neurobehavioural Sytems) and delivered using a Lenovo ThinkPad Edge laptop. Participants made their tapping responses using the spacebar key on the laptop (timing error of measurement system = ±0.92 ms).

#### 3.2.3 Analysis protocols

As stressed syllables occurred in the sentences every 500 ms (i.e. every other syllable), it was expected that participants would tap at this 2 Hz stress rate, producing 4 taps for each 8-syllable sentence. For example, for the sentence “Mary Mary quite contrary”, it was expected that participants would produce 4 taps in line with the syllables “Ma-”, “Ma-”, “quite” and “-tra-”, even though the metronome beats used when recording the sentences were not audible. Since there were 3 presentations of each sentence per trial, and tapping continued during the silent periods, participants could theoretically produce up to 20 taps per trial. However, since the sentence stimuli had no preceding beat, we expected that during the very first sentence presentation, participants would still be trying to find the rhythm. Consequently, they would only start to produce taps toward the end of the sentence rather than at the beginning. Therefore, when the trials were analysed, any taps produced during this very first presentation, and during the intervening silent periods were discarded. Only the 8 taps produced during the second and third presentations of each sentence were used.

Participants' tapping was analysed using three methods, following from three different theoretical approaches to representing rhythm in speech. In Method 1, inter-tap intervals (ITIs) were calculated to measure durational isochrony (periodicity) in tapping behaviour. As a second approach, the distances of participants' taps to rhythmic 'P-centres' in the speech stimuli were measured. P-centres are thought to be located near the onsets of vowels in stressed syllables (Allen, 1972; Morton et al., 1976). Note however that the exact location of the P-centre with respect to the vowel onset is influenced by the length of the initial consonant cluster of the syllable, and the length of the syllable coda (Allen, 1972; Morton et al., 1976; Port, 2003). Here, the vowel onset was used as a proxy marker of beat (P-centre) location for all the stressed syllables in the 4 nursery rhyme sentences, providing a consistent acoustic-phonetic landmark against which to assess tapping behaviour (rhythmic entrainment). The vowel onsets of the stressed syllables in each sentence were identified using Praat software (Boersma and Weenink, 2009) by an experienced rater, and participants' tap distance from each respective vowel onset was measured. The average of these distances for each participant was obtained, for each nursery rhyme sentence.

However, an important and inherent limitation of both ITI and ‘P-centre’ analyses is that rhythmic entrainment can only be measured at the rhythmic timescale or ‘tactus level’ of the observed motor response (here, at the 2 Hz or ‘stress’ rate). Since participants do not produce taps to *every* syllable or phoneme, there is insufficient information for measurement of syllable-level or phoneme-level entrainment. Therefore, these analysis methods are incapable of measuring rhythmic entrainment at timescales that are faster or slower than the motor response. However, *perceptually*, it is possible that listeners do entrain to more than one rhythmic timescale at the same time. For example, listeners could be timing their taps to the onset of the vowel within the syllable that is stressed. This would involve simultaneous rhythmic tracking at the *phoneme*-level to detect vowel onsets, the *syllable*-level to determine syllable boundaries, and the *stress*-level to determine the prominence status of each syllable. To analyse such potential *multi-timescale* perceptual entrainment, a novel approach is required.

Accordingly, the third and completely novel method we used to calibrate rhythmic behaviour measured participants' oscillatory phase of tapping with respect to amplitude modulation (AM) patterns in the speech signal. In our AM-based analysis, rather than requiring motor responses at multiple timescales, the speech signal itself was divided into multiple timescales. This enabled rhythmic entrainment to the AM cues at each timescale to be analysed separately. Specifically, we obtained Stress-, Syllable- and Phoneme-rate AMs from our nursery rhyme sentences, representing speech activity at prosodic, syllabic and phonetic timescales respectively on the basis of the modulation statistics of the speech itself. The Stress AM that is derived captures 'strong-weak' prosodic prominence patterns, the Syllable AM captures syllable units, and the Phoneme AM captures fast phoneme cues (such as plosive consonants). Each AM was extracted by band-pass filtering the speech amplitude envelope of the nursery rhyme sentences at the appropriate rate (Stress AM: 0.8–2.5 Hz, Syllable AM: 2.5–12 Hz, Phoneme AM: 12–40 Hz). For a more detailed description of the AM method, please see Appendix 1. For a discussion on the derivation and testing of these AM measures, please see Leong (2012).

**Fig. 3:**
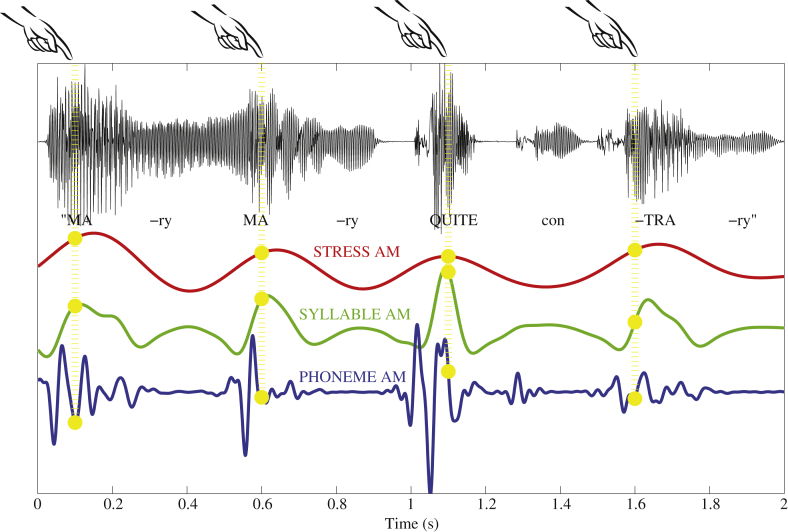
Example of AM-based phase analysis for the nursery rhyme sentence “Mary Mary quite contrary”. The acoustic waveform of the sentence is shown in black. The extracted Stress, Syllable and Phoneme AMs are shown in red, green and blue respectively below. Hand icons represent the hypothetical occurrence of the 4 taps. For each tap, the instantaneous AM phase at the point of occurrence (yellow dot) was measured.

As shown in Fig. 3 which depicts spectral band 3 of the S-AMPH model, these three rates produced oscillatory patterns on different timescales, ranging from slow (Stress AM, in red) to fast (Phoneme AM, in blue). To measure oscillatory entrainment to these speech AM patterns, we measured the *phase-locking* of the tapping responses of participants to each of the AM rates (Stress, Syllable, Phoneme). Our rationale was that if participants were timing their responses to the rhythmic information at that timescale, then all their taps should fall at the same oscillatory phase for the corresponding AM – i.e. responses should be consistently phase-locked to either the Stress AM, the Syllable AM or the Phoneme AM. Accordingly, for each tap, we measured the *instantaneous phase* of the Stress, Syllable and Phoneme AMs at the time when the tap was produced. Fig. 3 illustrates the rationale behind this method. Note that actual responses could in fact be timed to earlier or later AM cycles than those depicted in the Figure; this possibility is considered further when discussing the results.

The instantaneous oscillatory phase of each AM was derived from its analytic signal, which was computed using the Hilbert transform (Gabor, 1946). In this paper, oscillatory phase is considered to take values between −1π and 1π radians, where a value of 0π radians represents the peak of the oscillatory cycle and values of −1π and 1π radians equivalently represent the trough of the oscillatory cycle. For each tap, the instantaneous Stress AM, Syllable AM, and Phoneme AM phase in the spoken sentence was measured, resulting in 8 phase values for each AM rate, per sentence, per participant. The circular median of these 8 phase values was then taken, providing individual phase scores for each participant, at each respective AM rate, and for each sentence. To reduce the variability in the responses, phase scores for the two trochaic sentences (‘Mary Mary’ and ‘Simple Simon’) and the two iambic sentences (‘Queen of Hearts’ and ‘St Ives’) were averaged together to give mean trochaic and iambic phase scores. All analyses were performed in Matlab, and circular tests were conducted using the Matlab Toolbox for Circular Statistics (Berens, 2009).

### 3.3 Results

#### 3.3.1 Duration isochrony: using inter-tap intervals (ITIs) to calibrate rhythmic responding

**Fig. 4:**
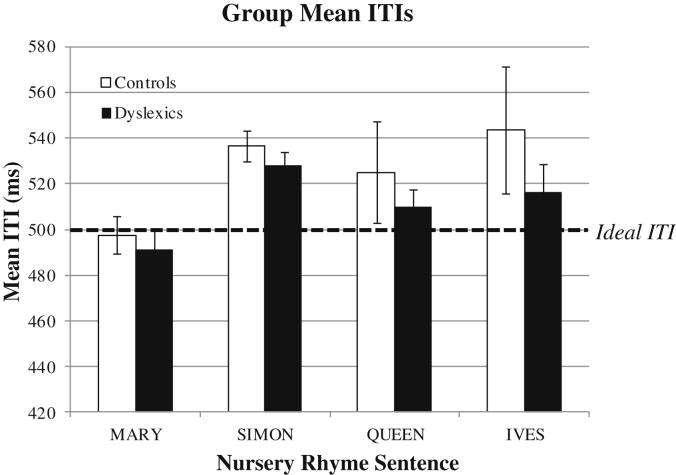
Group mean inter-tap interval (ITI) values for each nursery rhyme (Rhythmic calibration by ITIs). Controls are shown in white and dyslexics in black. Error bars show the standard error for each group. The dotted line indicates the ideal ITI of 500 ms (2 Hz).

It was expected that participants would generate inter-tap intervals (ITIs) of ∼500 ms, in line with the rate of stressed syllables in the sentences. As expected, on average over the 4 nursery rhyme sentences, the mean ITI was 525 ms for controls and 511 ms for dyslexics. Fig. 4 shows the group mean ITIs for each nursery rhyme sentence. As shown in Fig. 4, the ITIs produced for the nursery rhyme ‘Mary Mary’ were closest to the ideal 500 ms interval. For the other 3 nursery rhyme sentences, both control and dyslexic participants tended to produce longer ITI intervals than expected. From visual inspection of Fig. 4, it appears that dyslexics may be producing shorter ITIs than controls across the 4 nursery rhyme sentences. Accordingly, we tested for differences in ITIs between groups using a repeated measures ANOVA. For the ANOVA, Nursery Rhyme was the within-subjects factor and Group was the between-subjects factor. Results indicated that there was *no* significant effect of Group (*F*(1, 41) = 0.89, *p* = 0.35). However, there was a significant effect of Nursery Rhyme (*F*(3, 123) = 4.34, *p* < 0.01). Tukey HSD post-hoc analysis indicated that ITIs for the nursery rhyme ‘Mary Mary’ were significantly shorter than ITIs for ‘Simple Simon’ and ‘St Ives’ (*p* < 0.05 for both), but not for ‘Queen of Hearts’ (*p* < 0.05). Finally, there was no significant interaction between Group and Nursery rhyme (*F*(3, 123) = 0.30, *p* = 0.82), indicating that both controls and dyslexics were producing a similar pattern of ITIs across the 4 nursery rhymes.

The ITI only indicates the average rate of tapping, and not whether controls and dyslexics were early or late with respect to the actual stress beats (i.e. P-centres) in the acoustic signal. Participants who consistently tapped *before* the stress beat could produce the same ITI as participants who consistently tapped *after* the stress beat. To assess this directionality, participants' taps were analysed in relation to linguistic P-centre markers.

#### 3.3.2 P-centres: using tapping to vowel onsets to calibrate rhythmic responding

**Fig. 5:**
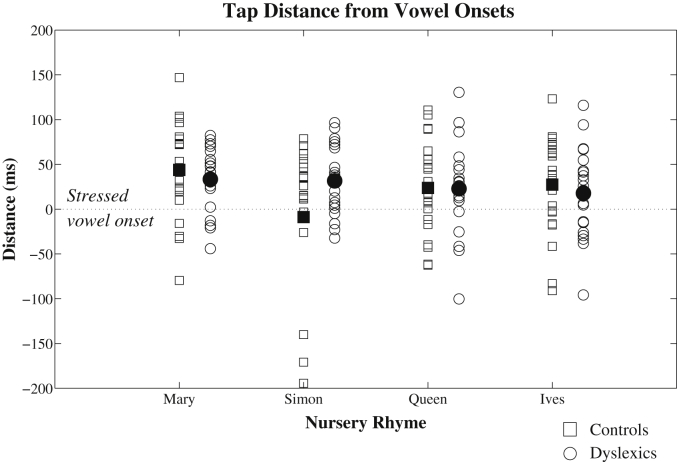
Distance of taps (in ms) from stressed vowel onsets in each nursery rhyme sentence (Rhythm calibration by P-centres). The horizontal dotted line at 0 ms indicates the location of stressed vowel onsets. Positive distance values indicate that taps occurred after the vowel onset. Negative distance values indicate that taps occurred before the vowel onset. Controls are shown as squares and dyslexics as circles. Open markers correspond to individuals (the average of 8 taps produced by that individual). Filled markers indicate the group means for each nursery rhyme sentence.

Fig. 5 shows the mean tapping distances measured for each individual with respect to ‘P-centres’ in the 4 nursery rhyme sentences. Individual participants are shown by separate markers, with controls as squares and dyslexics as circles. From visual inspection of Fig. 5, most of the participants tended to produce taps slightly *after* the actual onset of stressed vowels in the sentences (i.e. above the dotted line in Fig. 5). For the rhyme ‘Mary Mary’, the group mean distance was +43.9 ms for controls and +33.4 ms for dyslexics. For the rhyme ‘Simple Simon’, the group mean distance was −8.9 ms for controls and +31.6 ms for dyslexics. For ‘Queen of Hearts’, the group mean distance was +23.7 ms for controls and +22.8 ms for dyslexics. Finally, for ‘St Ives’, the group mean distance was +27.4 ms for controls and +17.8 ms for dyslexics.

To assess whether there were significant differences between controls and dyslexics in their tap distances to stressed vowel onsets, a repeated measures ANOVA was conducted. For the ANOVA, Nursery Rhyme was the within-subjects factor and Group was the between-subjects factor. Results indicated that there was again *no* significant effect of Group (*F*(1, 41) = 0.18, *p* = 0.67). The effect of Nursery Rhyme just missed significance (*F*(3, 123) = 2.65, *p* = 0.052). However, there was a significant interaction between Group and Nursery rhyme (*F*(3, 123) = 3.08, *p* < 0.05). Post-hoc analysis of this interaction using the Fisher LSD test indicated that there was a significant difference between controls and dyslexics for the nursery rhyme ‘Simple Simon’ (*p* < 0.05), but not for the other 3 nursery rhymes. However, inspection of Fig. 5 suggests that this apparent difference may be attributed to a few outlying control participants, rather than reflective of the group as a whole. Accordingly, we interpret the ANOVA results as indicating *no* consistent difference between controls and dyslexics in terms of tapping to P-centres in speech. Therefore, contrary to prediction, both the ITI (durational isochrony) and vowel onset (P-centre) analyses of tapping behaviour do not indicate any differences between controls and dyslexics in rhythmic entrainment to metrical speech. Finally, multi-timescale entrainment was examined using AM-based indices of phase-locking as generated by the novel AMPH method.

#### 3.3.3 AM phase-locking & tapping phase: calibrating rhythmic responding at multiple timescales using the S-AMPH model

**Fig. 6:**
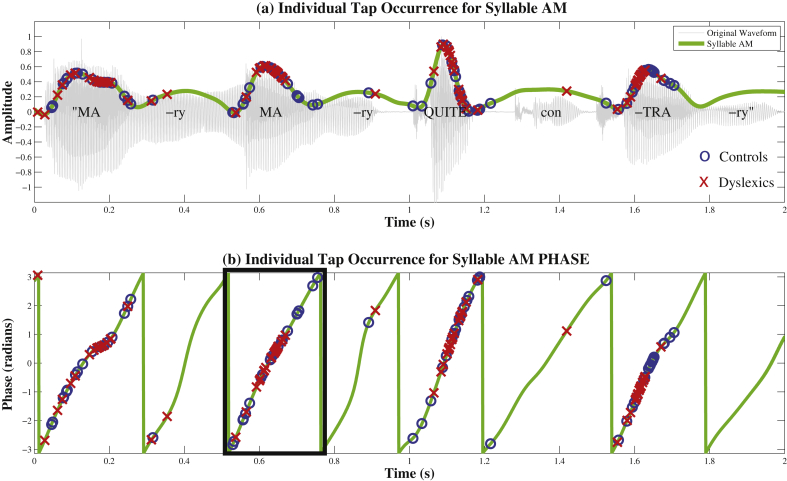
Example of participants' Syllable AM tapping phase, for a single presentation of the sentence “Mary Mary quite contrary” (Rhythm calibration by multi-timescale AMs). (a) Here, the actual time-course and distribution of participant taps (circles and crosses) is plotted with respect to the amplitude of the Syllable AM (green line). Each participant produces 4 taps, which occur around the 4 stressed syllables, “MA-”, “MA-”, “QUITE” and “-TRA” respectively. Note that each cycle (peak) of the Syllable AM corresponds to a single uttered syllable. Therefore the Syllable AM is an acoustic landmark for real speech events. (b) The same distribution of taps is now replotted with respect to the PHASE of the Syllable AM rather than its amplitude. Note that the peak of the Syllable AM corresponds to a phase value of 0 radians (mid-way up the *y*-axis), while the trough of the Syllable AM corresponds to phase values of −π/π radians. The circular equivalence of these two values explains the abrupt vertical cliffs in the phase plot. Note that the majority of participants' taps tend to occur within the temporal confines of a single AM cycle (−π to π radians), corresponding to the region of the increasing slope in the phase plot (solid black box).

**Fig. 7:**
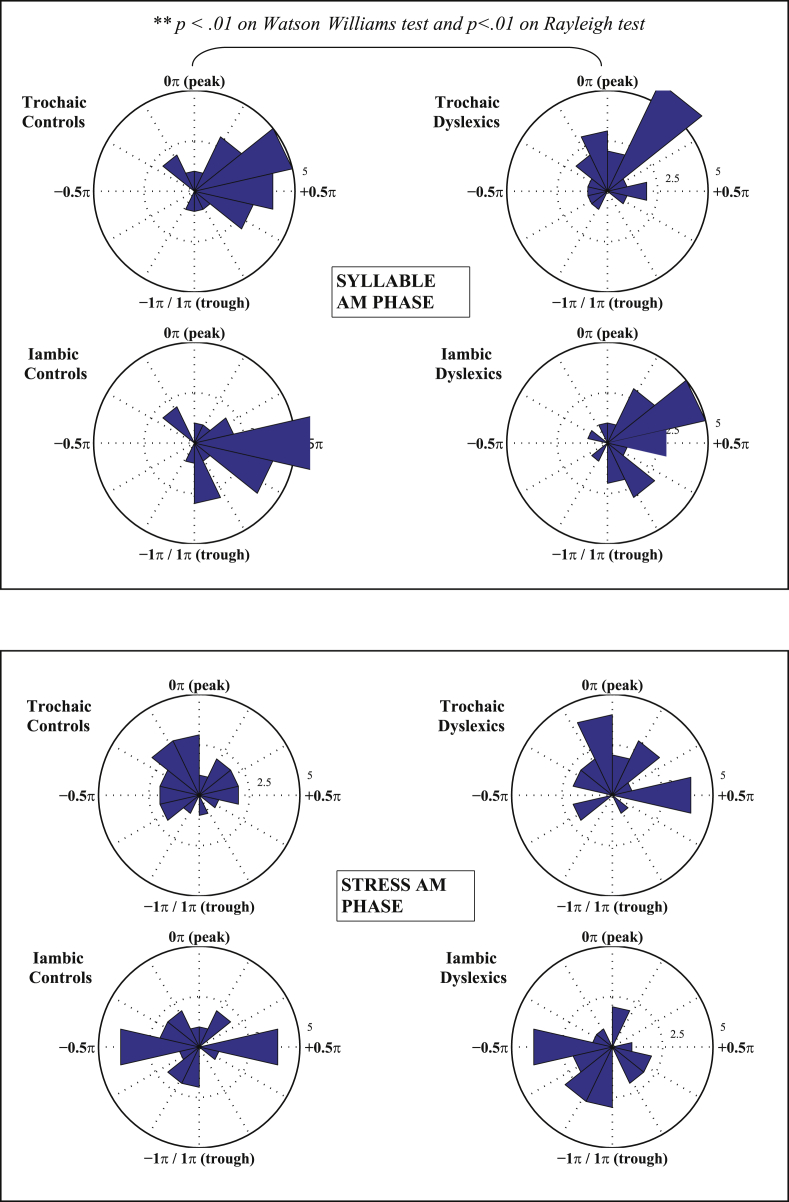
Compass phase plots show the distribution of tapping responses, binned with respect to Syllable AM phase (upper panel) and Stress AM phase (bottom panel). The top of the plot corresponds to the oscillatory peak, the bottom corresponds to the trough. Phase values increase in a clockwise direction. The length of radial spokes indicates the number of observations within each phase bin (with concentric circles indicating 2.5 and 5 observations). Controls are shown on the left and dyslexics on the right.

Table 1 shows the mean tapping phase (in radians) produced by each group for trochaic and iambic sentences, at Stress, Syllable and Phoneme AM rates. To illustrate the distribution of responses within each group for the Syllable rate AM, the actual timecourse of individual participants' taps is shown with respect to Syllable AM phase in Fig. 6, and summary histograms of these phase scores are shown as circular compass plots in Fig. 7. Taking the sentence “Mary Mary” as an example, Fig. 6a shows that the 4 taps that participants produced for this single presentation of the sentence were indeed close to the 4 stressed syllables in the utterance. Moreover, Fig. 6a illustrates that each Syllable AM cycle (or peak) does indeed correspond to a single uttered syllable in the sentence. Therefore the Syllable AM provides useful acoustic landmarks to real speech events. Accordingly, we henceforth interpret the rising phase of the Syllable AM (i.e. −π to 0 rad) as the rising/onset portion of the syllable, the peak of the Syllable AM (i.e. around 0 rad) as the approximate location of the vowel nucleus, and the falling phase of the Syllable AM (i.e. 0 to π rad) as the offset of the syllable.

In Fig. 7, the phase scores for control (left panel) and dyslexic (right panel) participants are plotted as compass phase plots at the Syllable and Stress AM rates, for trochaic and iambic sentences respectively. To produce these compass plots, the phase scores for each group were binned into 14 equal phase bins between −π and π radians. The number of participant scores falling within each phase bin is reflected in the radial length of the spokes in each compass plot. The rotational angle of the spokes reflects the circular phase of tapping. Strong clustering around a single spoke indicates phase-entrained responding at the group level. The top of the plot indicates the peak of the oscillatory cycle and the bottom indicates the trough, with phase values increasing in a clockwise direction. Consequently, the left half of the circular plot corresponds to the rising portion of the oscillatory cycle (e.g. syllable onset for the Syllable AM) whereas the right half of the circular plot corresponds to the falling portion of the oscillatory cycle (e.g. syllable offset for the Syllable AM). Thus the top right quadrant of each Syllable AM plot (phase values between 0π to +0.5π radians) corresponds to taps produced just after the oscillatory peak, when the cycle has just begun to descend. From visual inspection of the compass plots in Fig. 7, it appears that dyslexic participants may be producing taps at an *earlier* Syllable AM phase than controls, since the dominant rotational angle of their plots is shifted counter-clockwise, as compared to controls. Further, dyslexics appear to show stronger clustering for Stress AM phase, suggestive of multi-timescale entrainment.

To test whether there were statistically-significant differences between groups in terms of their Stress-, Syllable- and Phoneme AM phase of tapping, a two-step testing procedure was employed. First, we tested whether the (unbinned) phase values within each group and condition (trochaic/iambic) showed a significant concentration about a particular phase-value, rather than being uniformly distributed at all phase values. If participants were consistently timing their taps to a particular AM phase (i.e. phase-locking their responses), this should produce a narrow concentration of responses about a particular AM phase value (indicating strength of phase locking). Conversely, if no significant phase-locking was occurring (i.e. participants were all tapping at different phases), then the resulting phase distribution would be uniform. In this case, a further comparison of group means would not be meaningful since these means would not reflect a central tendency. To test for non-uniformity in the phase distribution, a Rayleigh circular test was used. The results of the Rayleigh test indicated that significant phase-locking was observed in *both* groups only with respect to **Syllable AM phase** (trochaic sentences: *z* = 8.1, *p* < 0.01 for controls, *z* = 5.9, *p* < 0.01 for dyslexics; iambic sentences: *z* = 8.4, *p* < 0.01 for controls, *z* = 5.9, *p* < 0.01 for dyslexics). Dyslexics also showed significant phase-locking to the **Stress AM** for trochaic sentences (*z* = 4.6; *p* < 0.01) but this missed significance for iambic sentences (*z* = 2.6; *p* = 0.07). Control participants did not show significant phase-locking to the Stress AM in either condition (trochaic: *z* = 2.0, *p* = 0.13; iambic: *z* = 0.6, *p* = 0.53). Neither group showed significant phase-locking to Phoneme AM phase in either trochaic or iambic sentences (*p* > 0.14 for all conditions). Therefore, further group comparisons were not conducted for the Stress AM or the Phoneme AM, since one or both groups had failed to show significant phase-locking at these two rates.

**Fig. 8:**
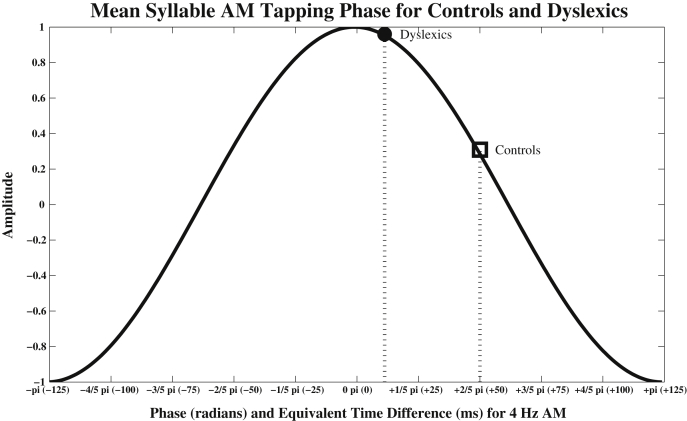
Illustration of the entrained Syllable AM phase for dyslexics as compared to controls, for TROCHAIC sentences. The *x*-axis shows the oscillatory phase of the AM cycle from −π to +π radians, and the equivalent time difference for a 4 Hz AM cycle. The group mean tapping phase for controls (open square) and dyslexics (filled circle) are annotated on the AM cycle, showing that dyslexics are entrained 0.31π radians or 39 ms earlier on the Syllable AM cycle.

Having established that significant phase-locking was occurring with respect to the Syllable AM in both groups, we proceeded to test whether the mean *angle* of Syllable phase-locking was the same or different between groups. From Fig. 7, it appears that dyslexics may be phase-locking to an *earlier* angle of the Syllable AM as compared to controls. Accordingly, we applied the Watson–Williams test (a circular analogue of the ANOVA) to test for group differences in mean entrained Syllable phase. The results of the Watson–Williams test showed that there was indeed a significant difference between groups for Syllable AM phase in *trochaic* sentences (*F*(1,42) = 7.88, *p* < 0.01), but not for iambic sentences (*F*(1,42) = 0.64, *p* = 0.43). Therefore, dyslexics were entraining their taps to a significantly *earlier* Syllable AM phase in trochaic sentences as compared to controls. For the current Syllable rate of 4 Hz, the mean phase difference between groups of 0.31π radians was equivalent to a time difference of ∼39 ms. This group difference in entrained Syllable AM phase (and the equivalent time difference) is illustrated in Fig. 8.

Due to the small number of trials in our experiment, there was concern that participants' tapping responses may not be significantly phase-locked at the *individual* level, precluding further *group* level analysis. Therefore, we performed similar analyses at the individual level, to test whether the tapping responses of individual participants were also significantly phase-locked. For the Syllable AM and trochaic sentences, 11 controls and 13 dyslexics showed significant phase-locking, and for the iambic sentences 9 controls and 7 dyslexics showed significant phase locking. When the group analyses were restricted to only those individuals who showed significant phase locking, the same results were found as for the whole-group analyses (i.e. dyslexics tapped at a significantly earlier phase for the trochaic sentences, *p* < 0.05, but not the iambic sentences, *p* > 0.10).

#### 3.3.4 Correlation between AM tapping phase, literacy and phonology

Finally, we wanted to test if individual differences in preferred AM phase of tapping would be related to individual differences in reading, spelling and phonology. Accordingly, we performed circular–linear correlations between participants' Stress, Syllable and Phoneme AM phase of tapping for both trochaic and iambic sentences, and their performance on the reading, spelling and Spoonerisms tasks. Correlations were performed in three ways: (1) across all subjects, (2) across controls only, and (3) across dyslexics only. The results are shown in Table 2, which provides the correlation coefficients (indicative of effect size) for each comparison. The marked *p*-values are uncorrected for the 54 comparisons conducted (as this would lead to a loss of power and increased Type II error), and should be interpreted with caution. Correlations across all subjects indicated that individual differences in Syllable phase of tapping for trochaic sentences were strongly related to participants' performance in Spelling (*r* = 0.52, *p < 0.01*). Although this correlation between Syllable tapping phase and Spelling was lower when conducted *within* dyslexic and control groups, both groups still showed moderately high correlation values (*r* = 0.30 for controls, *r* = 0.42 for dyslexics), indicating a similar pattern of performance in both groups. Still considering Syllable tapping phase, there was also a relatively strong relationship with phonology (Spoonerisms, *r* = 0.35, *p* = 0.07) across all participants, but this effect appeared to be driven by the dyslexic group (*r* = 0.35, *p* = 0.07) rather than the control group (*r* = 0.06, ns). For Stress phase of tapping, there was a relationship between tapping in iambic sentences and phonology across all participants (*r* = 0.39, *p* ≤ 0.05). This effect appeared to be very strongly driven by the dyslexic group (*r* = 0.67, *p* < 0.01), consistent with the significant phase-locking observed in dyslexics to the Stress AM. No significant correlations were observed for Phoneme AM phase across all participants (although there was a trend in the dyslexic group for Iambic phase and reading), consistent with the non-phase-locked distributions found for this temporal rate.

### 3.4 Interim summary & discussion

Here we used 3 methods to calibrate the accuracy of rhythmic responding, two traditional methods (inter-tap interval, P-centres) and one novel method, a multi-timescale analysis based on the S-AMPH model. In the *ITI analysis*, both control and dyslexic participants showed evidence of appropriate rate entrainment, generating ITIs that were close to the 2 Hz stress rate of the sentences. In fact, dyslexics were even closer than controls to the ideal ITI rate (511 ms compared to 525 ms), although this group difference was not statistically significant (possibly due to the small number of trials in our experiment). In the *P-centre analysis* (tap to vowel onsets), both control and dyslexic participants appeared to be timing their taps to occur just after the onsets of stressed vowels in the sentences. Although control participants appeared to be tapping slightly earlier than dyslexics for the nursery rhyme ‘Simple Simon’, this effect appeared to be driven by a few outlying participants, and there were no consistent differences between groups in timing on a P-centres analysis. Again this lack of difference could have been due to the low trial number in our experiment. Finally, *multi-timescale AM analysis* using the S-AMPH model revealed significant differences in dyslexic entrainment at the Syllable (∼4 Hz) and Stress (∼2 Hz) tactus levels. For trochaic sentences, dyslexics were consistently entrained (phase-locked) to an *earlier* portion of the Syllable AM cycle, as measured by phase angle, compared to controls. Therefore, dyslexics were producing taps that were highly regular in interval, but overall shifted earlier in time with respect to the speech signal. Moreover, individual differences in participants' preferred Syllable phase of tapping was found to be related to their spelling ability, and also related to their phonological awareness. Further, dyslexic participants showed significant entrainment to the Stress AM, whereas controls did not. Dyslexic participants showed significantly non-uniform phase distributions for the trochaic sentences, and this measure was almost significant for the iambic sentences (*p* = 0.07). For iambic sentences, Stress AM phase was significantly related to phonological awareness. It is interesting that group differences did not always occur for *both* trochaic and iambic sentences. For example, differences in Syllable AM phase of tapping were observed for trochaic but not iambic sentences. Given that significant phase-locking was observed for *both* types of sentences, the iambic lack-of-effect was not due to greater response variability (noise). The trochaic pattern occurs more frequently in English words than the iambic pattern (Cutler and Carter, 1987), and therefore receives preferential processing even from infancy (Jusczyk et al., 1999, 1993). The phase difference we observe for trochaic sentences (but not iambic sentences) could therefore reflect differences in prosodic development in dyslexics.

Since neuronal oscillatory activity can affect behavioural response patterns (Henry and Obleser, 2012), dyslexics' different entrained phase-of-tapping could indicate differences in their underlying neuronal oscillatory activity. A relationship would also be expected on the temporal sampling account of the phonological deficit in dyslexia proposed by Goswami (2011). According to the temporal sampling framework, atypical neuronal entrainment to lower temporal frequencies of modulation in the speech signal (delta and theta, <10 Hz) is one cause of the atypical development of phonological representations for words in the mental lexicon found in developmental dyslexia across languages. Dyslexics' ‘additional’ Stress AM phase-locking as revealed here by tapping could be compensatory – dyslexics might be using an additional source of temporal information to help them to find and entrain to the beat, to compensate for their inaccurate Syllable AM entrainment. This interpretation suggests that whereas controls only needed to keep track of the Syllable AM in order to find the beat in a sentence, dyslexics had to track *both* the Stress AM and the Syllable AM in order to produce a stable tapping response. Further research is required to test this interpretation.

## 4 Experiment 2: speech rhythm production

### 4.1 Rationale

Group differences in speech rhythm perception in Experiment 1 were revealed by multi-timescale AM analysis but not by more traditional methods of rhythm calibration (ITIs, P-centres). In order to investigate whether group differences in AM entrainment would also occur in speech production, a second experiment was conducted. Here we examined whether there would be differences in the production of speech rhythm between control and dyslexic participants. Accordingly, we asked participants to produce sentences in a rhythmic manner, in time to a pacing metronome beat. To facilitate rhythmic production, sentences with a strong and regular rhythmic template (nursery rhymes) were used.

### 4.2 Methods

#### 4.2.1 Materials

The same four nursery rhyme sentences used in Experiment 1 were used. Each sentence was 8 syllables in length and had a binary alternating rhythm of strong (s) and weak (w) syllables. Two sentences (“Mary Mary quite contrary” and “Simple Simon met a pieman”) had a trochaic stress pattern while the other two sentences (“as I was going to St Ives” and “the Queen of Hearts she made some tarts”) had an iambic stress pattern.

#### 4.2.2 Task description

Participants were asked to recite each of the four nursery rhyme sentences aloud, speaking in time to a 2 Hz metronome beat. As we were interested in testing rhythmic entrainment (i.e. synchronisation of speech patterns to an external beat), participants were instructed to follow the beat of the metronome, rather than setting their own speaking rate. This design was inspired by the ‘speech cycling’ paradigm used by Cummins and Port (1998). Participants repeated each sentence five times before moving on to the next sentence. The metronome beat was presented binaurally using Microsoft LX-3000 headphones, at a sound level that was comfortable for participants. Speech productions were simultaneously recorded using the built-in microphone. Participants were allowed to practice producing the sentences in time to the beat beforehand, and the recording commenced only after they indicated that they were satisfied that they could produce the sentences successfully. Participants produced the trochaic sentences first (‘Mary Mary’ and ‘Simple Simon’) followed by the iambic sentences (‘Queen of Hearts’, ‘St Ives’).

#### 4.2.3 Analysis protocols

Our aim was to measure the rhythmic *control* of participants when actively constraining or synchronising their speech patterns to a rhythmic template (the metronome). Therefore, the analysis measured the degree of rhythmic synchronisation present in the speech signal of control and dyslexic participants using two measures. First, to measure *external* rate synchronisation (or synchronisation to the external pacing beat), the durational interval between successive vowel onsets was computed. Second, to measure *internal* synchronisation between speech units at different timescales, AM cross-frequency phase-locking indices were computed using the S-AMPH (spectral band 1). Each of the four nursery rhyme sentences was analysed separately. Although each sentence was repeated 5 times, only the last 3 repetitions were used in the analysis, as participants achieved a more stable speaking rhythm in these later utterances.

##### 4.2.3.1 External rate synchronisation: vowel onset intervals (VOIs)

For this analysis, the interval between syllable vowel onsets was taken as a proxy indicator of syllable length, and therefore syllable rate.^1^ If participants were successfully synchronising their rate of syllable production to the external pacing beat, it was expected that their produced vowel onset intervals (VOIs) would be similar to the metronome beat interval (or would be integer subdivisions of this interval). To determine individual VOIs, for each spoken sentence, the onsets of the 8 syllable vowel nuclei were manually located using Praat software. From the timing of these 8 vowel onsets, VOIs were computed by subtracting the time of the current vowel onset from the time of the next vowel onset, resulting in 7 VOIs. These 7 VOIs were then averaged (across the 7 intervals and 3 sentence repetitions) to produce a mean VOI for each participant and nursery rhyme sentence. The resulting mean VOI was analogous to the inter-tap interval (ITI) computed for the tapping data in the perception experiment.

The time difference between vowel onsets and metronome beats was *not* used as a measure because in some cases (e.g. iambic rhymes), the pace of participants' utterances was quite different from the metronome rate. This led to ambiguities in determining which vowel onset corresponded to a given metronome beat. Also, if participants were producing syllables at regular intervals, but at a different rate from the metronome, the time difference between vowel onsets and metronome beats would change as the utterance progressed. Measurement of these time differences would indicate that the utterance was not rhythmically-regular, when in fact the utterance was regular, but with a different pulse rate from the metronome. Therefore, in the VOI analysis, syllable vowel rate was measured instead of the absolute vowel-to-metronome time difference.

##### 4.2.3.2 Internal synchronisation: AM cross-frequency phase-locking

To measure internal (within-speech) rhythmic synchronisation, cross-frequency phase-locking measures were applied. First, the Stress, Syllable and Phoneme AMs were extracted from the speech samples produced by the control and dyslexic participants using the AM-extraction procedure in the S-AMPH model described in Appendix 1. Two cross-frequency phase-locking measures were then computed. These were (a) the *strength* and (b) the *angle* of phase-locking (synchronisation) between the various AM patterns in speech. Since the AMs represent different speech units within the linguistic prosodic hierarchy (e.g. prosodic stress feet, syllables, phonemes), this analysis also examines the temporal dynamics of prosodic organisation in speech.

To compute the *strength* of synchronisation between pairs of speech AMs (e.g. Stress:Syllable AM), an *n:m* phase synchronisation index (PSI) was computed. The *n:m* phase-locking measure was originally conceptualised by Tass et al. (1998) to quantify phase-synchronisation between two oscillators of different frequencies, where the oscillators could represent muscle activity or neural activity. This measure was subsequently adapted for use in neural analyses of oscillatory phase-locking (e.g. Schack and Weiss, 2005; Kralemann et al., 2007, 2008), and we apply this adaptation to our speech AMs here. The PSI was computed as:

(1) $$\text{PSI}=\left|\langle e^{i\left(n\theta_{1}-m\theta_{2}\right)}\rangle \right|$$

In Equation (1), *n* and *m* are integers describing the frequency relationship between the two AMs being compared. For the Stress:Syllable AM comparison, an *n:m* ratio of 2:1 was used because we had a priori knowledge that the stress rate of the sentences was half that of the syllable rate (i.e. both trochaic and iambic sentences contain stress every 2 syllables). For the Syllable:Phoneme AM comparison, we had no strong a priori rationale for selecting one *n:m* ratio over another since the syllables could contain different numbers of phonemes. Therefore, we computed PSI scores across a variety of possible *n*:*m* ratios (2:1, mean PSI = 0.07, s.d. = 0.003; 3:1, mean PSI = 0.22, s.d. = 0.007; 4:1, mean PSI = 0.08, s.d. = 0.003; 5:1, mean PSI = 0.06, s.d. = 0.003). The *n:m* ratio of 3:1 clearly yielded the highest overall PSI score (0.22), and so we took this to indicate that 3:1 was the most dominant phase-locking ratio between the Syllable and Phoneme AMs in our stimuli. Accordingly, we used this ratio in further analyses. θ_1_ and θ_2_ refer to the instantaneous phase of the two AMs at each point in time. Therefore, (*n*θ_1_ – *m*θ_2_) is the generalised phase difference between the two AMs, which is computed by taking the circular distance (modulus 2π) between the two instantaneous phase angles. The angled brackets denote averaging of the complex exponential function of this phase difference over all time-points. The PSI is the absolute value of this average, and can take values between 0 (no synchronisation) and 1 (perfect synchronisation). PSI values were computed for control and dyslexic groups, and for trochaic and iambic sentences.

To compare the *angle* of cross-frequency phase-locking between groups, the generalised phase-difference (*n*θ_1_ – *m*θ_2_) from Equation (1) was used. This effectively converts the two oscillators to the same frequency while retaining any phase differences. If two oscillators of the same frequency are perfectly in phase, they will have a constant phase-difference of 0π radians at all time-points, meaning that peaks and troughs will occur at exactly the same time in both oscillators. By contrast, if two oscillators are perfectly out-of-phase, then a peak in one oscillator will coincide with a trough in the other oscillator, giving a constant phase-difference of 1π radians at all time-points. In the phase angle analysis, we measured the mean generalised phase difference between pairs of AMs (Stress:Syllable and Syllable:Phoneme), for trochaic and iambic sentences, for both control and dyslexic groups. If dyslexics showed a different angle of phase-locking between AMs (as evidenced by a smaller or larger phase-difference), this would suggest that their speech had a different temporal hierarchical organisation as compared to controls. To check that the phase values observed within each group were sufficiently concentrated (i.e. non-uniformly distributed) to enable the comparison of group means, a Rayleigh test was first performed. Subsequently, a Watson–Williams test was used to assess whether control and dyslexic groups showed the same angle of phase-locking between Stress & Syllable AMs, and between Syllable and Phoneme AMs.

### 4.3 Results

**Fig. 9:**
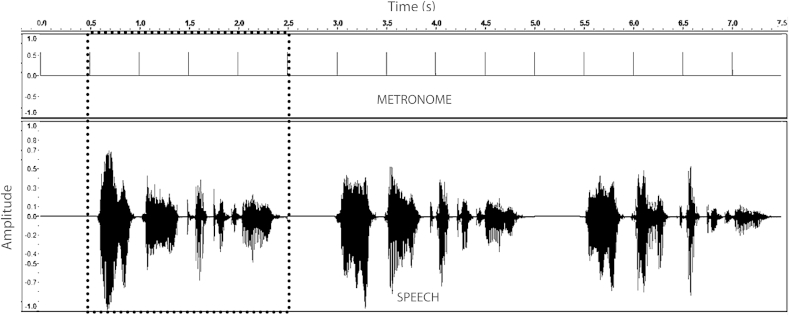
Example of the nursery rhyme sentence “Mary Mary quite contrary” produced by a dyslexic participant, uttered three times. The vertical tick marks in the top part of the figure indicate the pacing metronome beats. The bottom part of the figure shows the waveform of the utterance. Each iteration of the sentence (8 syllables) was spoken to fit within 4 metronome beats (dotted box).

The vast majority of participants spontaneously produced two syllables per metronome beat instead of one syllable per beat, although they were not explicitly instructed to do so. Fig. 9 shows an example of an utterance produced by a dyslexic participant, who produced a sentence of 8 syllables to fit within 4 metronome beats. This example suggests that participants preferred to impose a regular *stress* rate on their utterances and not a regular *syllable* rate. Participants preferred to time every alternate (stressed) syllable to the beat, instead of every syllable. This behaviour is consistent with the proposal that English is a stress-timed language (Abercrombie, 1967; Pike, 1945). However, it is also possible that participants chose this faster 4 Hz syllable rate of speaking (as compared to a 2 Hz syllable rate) because it was closer to their spontaneous speaking rate. A few participants (2 controls and 2 dyslexics) spontaneously chose to produce 1 syllable per beat instead of 2 syllables per beat, and were consistent in using this slower rate of production across all 4 sentences. Therefore, the rate preference of participants did not seem to differ between groups. A further 3 controls also used this slower rate of production for 1 or 2 out of the 4 sentences. All of these more slowly-produced ‘syllable-timed’ utterances were excluded from the analysis.

#### 4.3.1 External rate synchronisation: vowel onset intervals (VOIs)

**Fig. 10:**
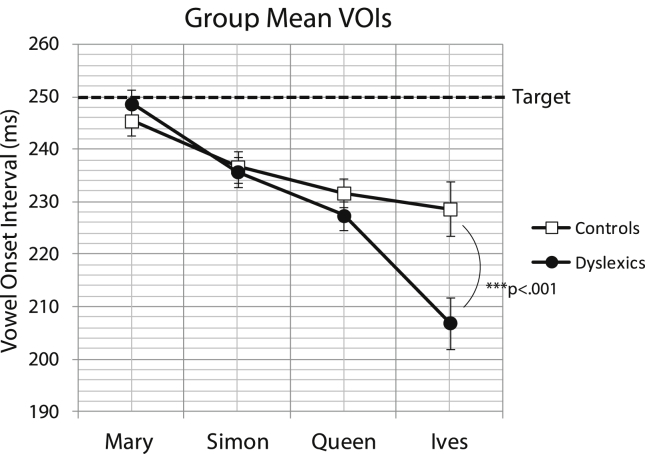
Mean Vowel Onset Intervals (VOIs, in ms) for each nursery rhyme and group. The ideal target interval was 250 ms, this is marked on the graph with a dotted line. Controls are shown as squares and dyslexics as circles. Error bars indicate the standard error.

Since the time interval between metronome beats was 500 ms (2 Hz), and participants uttered 2 syllables per beat (4 Hz), we expected that their vowel onset intervals would be close to 250 ms. As shown in Fig. 10, both control and dyslexic participants indeed produced VOIs that were close to 250 ms for the trochaic nursery rhyme ‘Mary Mary’. However, for the iambic nursery rhymes ‘Queen of Hearts’ and ‘St Ives’, VOIs grew shorter for both groups. The dyslexics, in particular, shortened their VOIs for ‘St Ives’ drastically to under 210 ms on average. To analyse whether there were significant group differences in VOI, a repeated measures ANOVA was conducted with Nursery Rhyme as the within-subjects factor, and Group as the between-subjects factor. As expected, there was a significant main effect of Nursery Rhyme (*F*(3,102) = 39.2, *p* < 0.0001), with vowel intervals getting significantly shorter in a graded fashion from ‘Mary Mary’ to ‘Simple Simon’ to ‘Queen of Hearts’ to ‘St Ives’. There was no main effect of Group (*F*(1,34) = 2.62, *p* = 0.15), but there was a significant interaction between Nursery Rhyme and Group (*F*(3,102) = 7.84, *p* < 0.0001) indicating that controls and dyslexics differed in their pattern of performance across the 4 nursery rhymes. To investigate this interaction further, a Tukey HSD post hoc test was conducted. Results of the post-hoc test revealed significant differences between groups only for the iambic nursery rhyme ‘St Ives’ (*p* < 0.001). This difference is marked on the graph in Fig. 10. Therefore, both controls and dyslexics showed poorer external synchronisation (shorter VOIs) for iambic as compared to trochaic sentences, but in addition dyslexics showed significantly worse external rate synchronisation than controls for the iambic nursery rhyme ‘St Ives’.

#### 4.3.2 Internal synchronisation: AM cross-frequency phase-locking

*Strength of Phase-Locking (Phase Synchronisation Index, PSI)*. As explained above, phase locking between different AM rates is computed as an index ranging from 0 to 1, with 1 indicating perfect phase locking. For the Stress:Syllable AM pair, the mean phase synchronisation index (PSI) for controls was 0.18 for trochaic sentences and 0.21 for iambic sentences. For dyslexics, the PSI values were slightly higher, at 0.22 and 0.24 for trochaic and iambic sentences respectively. For the Syllable:Phoneme AM pair, control PSI scores for trochaic and iambic sentences were 0.22 and 0.22 respectively. For dyslexics, the PSI scores were similar, at 0.20 and 0.23 respectively. Independent samples *t*-tests comparing these respective PSI scores indicated that there were *no* significant group differences for any comparison (*p* > 0.10 for all 4 comparisons). Therefore, dyslexics and controls showed an equal strength of phase-locking for both AM pairs (Stress:Syllable; and Syllable:Phoneme), and for both trochaic and iambic sentences.

*Angle of Phase-Locking*. For both Stress:Syllable and Syllable:Phoneme AM pairs, both groups produced highly-concentrated phase difference values (*p* < 0.0001 for all comparisons and groups on the Rayleigh test). Therefore, further tests comparing group means were justified. For the Stress:Syllable AM pair, the mean angular phase difference for controls was 0.11π radians (trochaic) and 0.05π radians (iambic). For dyslexics, the mean phase difference was slightly smaller, at 0.08π radians (trochaic) and 0.03π radians (iambic). However, Watson–Williams tests indicated that groups did *not* differ in their angle of phase-locking for the Stress:Syllable AM pair for either trochaic or iambic sentences (trochaic: *F*(1,38) = 0.19, *p* = 0.67; iambic: *F*(1,36) = 0.14, *p* = 0.71).

**Fig. 11:**
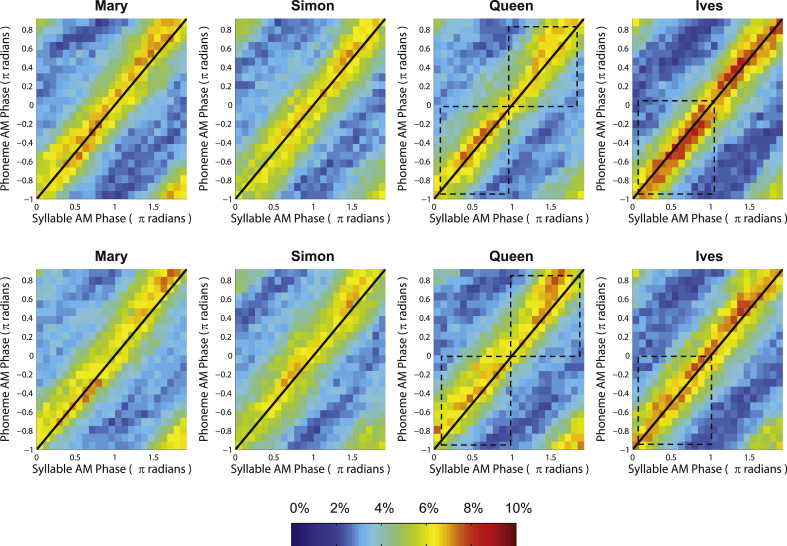
Syllable:Phoneme AM (*n*:*m* = 3:1) phase distributions for groups and sentences. The *x*- and *y*-axes show phase values for the Syllable AM (0 to 2π radians) and Phoneme AM (−π to π radians) respectively. The pixel colour indicates the frequency of occurrence (%) for each Phoneme AM phase value (*y*-axis), with respect to the concurrent Syllable AM phase value (*x*-axis), computed over all time-points, and averaged across participants. Blue colour indicates low percentage of occurrence and red colour indicates high percentage of occurrence. The majority of observations lie close to the diagonal black line, which indicates an (*n*θ_1_ – *m*θ_2_) difference of 1π radians. For both axes, phase values have been binned into 24 bins so that each bin (pixel) has a width of 0.08π radians. The 4 columns show the 4 different nursery rhyme sentences. Controls are shown in top row, dyslexics in bottom row. Dyslexics' Syllable:Phoneme phase distribution is consistently shifted upwards relative to controls by ∼1 bin. This is especially noticeable for iambic sentences (examples highlighted in boxes).

For the Syllable:Phoneme AM pair, the mean phase difference for controls was 0.94π radians (trochaic) and 0.95π radians (iambic). For dyslexics, the mean phase differences were again smaller, at 0.88π radians (trochaic) and 0.88π radians (iambic). This time, Watson–Williams tests indicated that there was a highly significant difference between groups for *iambic* sentences (*F*(1,36) = 7.7, *p* < 0.01), while the group difference for trochaic sentences just missed significance (*F*(1,38) = 3.9, *p* = 0.055). To illustrate this group difference in the Syllable–Phoneme phase-locking angle, the Syllable:Phoneme (*n*:*m*) phase distribution for each nursery rhyme by group is shown in Fig. 11 (controls top row, dyslexics bottom row). For all sentences, the majority of observations (i.e. time-points) lie close to the diagonal line indicating a 1π radians (*n*θ_1_ – *m*θ_2_) phase difference between the Syllable AM and the Phoneme AM. For the two iambic sentences ‘Queen’ and ‘St Ives’, the dyslexic phase distribution is shifted upwards by ∼1 bin width (equivalent to 0.08π radians), consistent with their slightly smaller Syllable:Phoneme (*n*θ_1_ – *m*θ_2_) phase difference.

### 4.4 Interim summary & discussion

In the vowel onset interval analysis (external rate synchronisation), both controls and dyslexics showed poorer synchronisation for iambic sentences than for trochaic sentences, producing shortened VOIs indicative of increased speaking rate. It is possible that this effect reflected a change in strategy by participants as the rhymes increased in metrical complexity. For example, for the simpler trochaic rhymes, participants could have been trying to entrain each of the 4 stressed syllables to the external beat. As the metrical complexity of the rhymes increased, possibly taking up more cognitive resources, participants could have switched to a simpler strategy of timing only 1 or 2 of the 4 stressed syllables to the metronome beat, while disregarding the timing of the intervening unstressed syllables. These unstressed syllables would then contract in duration, producing the observed decrease in VOIs. Nevertheless, the data indicate that both groups used similar strategies.

The iambic nursery rhymes were metrically more complex, and in particular, the nursery rhyme ‘St Ives’ appeared to be metrically more complex than the other three nursery rhymes. Many participants were unsure of how to assign stress on the first three syllables “*As I was…*”. According to the original nursery rhyme, these syllables should have been spoken with a ‘w–s–w’ pattern. However, a significant number of participants in both control and dyslexic groups chose to produce a ‘s–w–w’ pattern instead for these first three syllables (e.g. “*AS i was…*”). Despite this difficulty in metrical patterning, controls still maintained adequate rhythmic synchronisation to the external beat, producing syllables close (∼20 ms) to the target interval of 250 ms (i.e. an error of 8.5%). However, dyslexics were less able to maintain rhythmic synchronisation in the face of metrical complexity, producing syllables that were significantly shorter than controls (∼40 ms shorter than the target interval, or an error of 17.2%). Therefore, our results indicate that dyslexics' rhythmic synchronisation to *external timing* is less stable and more prone to breakdown under increased task complexity or mental load.

The second set of analyses considered internal timing, using AM cross-frequency phase-locking measures to reveal *internal* speech synchronisation at multiple timescales simultaneously. Regarding the strength of phase-locking as measured by the PSI, both controls and dyslexics showed equally strong internal phase-locking between Stress and Syllable AMs, and between Syllable and Phoneme AMs. Regarding the timing of phase locking however, a significantly different *angle* of phase-locking between Syllable and Phoneme AMs was observed for dyslexic participants. This result is similar to that observed in the rhythm perception (tapping) experiment. In both cases, the *strength* of dyslexic entrainment (phase-locking) per se was similar to controls, but the *angle* of phase-locking was different. This indicates that the temporal co-ordination of the ‘prosodic hierarchy’ is different in dyslexia. Both the perception and production experiments revealed that atypical dyslexic phase-locking involved the Syllable AM rate.

At first glance, it may not be apparent to the reader why the internal synchronisation between speech AMs should change as result of synchronisation to an external metronome beat. However, as noted by Cummins and Port (1998), rhythm comprises more than recurrent isochronous periods. Rather, rhythm can be viewed as the perceptual product of a hierarchically-nested temporal structure, with temporal constraints operating across multiple levels. This definition of rhythm is conceptually related to that of metrical phonology (Liberman and Prince, 1977), which also proposes the existence of multiple hierarchically-nested levels of rhythmic organisation in language, exemplified by syllables and stress feet. Accordingly, when one is speaking in time to an external beat, it follows that the speaker would attempt to align his or her spoken phonological hierarchy (i.e. of syllables, stress feet, etc), with the perceived (or idealised) rhythmic hierarchy of the entraining beat pattern. This hierarchical alignment was elegantly demonstrated by Cummins and Port (1998) in their original speech cycling experiment, where participants' production of stressed syllables within a phrase (e.g. “BEG” and “DIME” in the phrase “BEG for a DIME”) was rhythmically constrained to fall within certain phase regions of the over-arching phrase cycle. Therefore, misalignments in dyslexics' *produced* phonological hierarchy (as indexed by AM cross-frequency synchronisation) could indicate differences in the way dyslexics *perceive* the hierarchical structure of rhythm, differences in the way their mental phonological representations are hierarchically-organised, or differences in the way their motor articulators are *co-ordinated* to produce the rhythmic utterance. More research is required to adjudicate between these explanations. In the following discussion, we explore the organisation of mental phonological representations in dyslexia further, as atypical phonological representation is considered to be the cognitive hallmark of dyslexia across languages (Ziegler and Goswami, 2005), and was the original motivation for the temporal sampling theory.

## 5 Final discussion & conclusion

### 5.1 Dyslexia is associated with abnormal rhythmic entrainment to syllable patterns in speech

Here, we investigated rhythmic entrainment to nursery rhyme speech in dyslexia, examining both rhythm *perception* and *production*, and using both traditional interval- and P-centre based analyses and a novel multi-timescale analysis method based on amplitude modulation phase hierarchies (AMPHs, Leong, 2012). Given recent proposals regarding atypical neuronal oscillatory entrainment in dyslexia (Goswami, 2011; Lehongre et al., 2011; Hämäläinen et al., 2012b), we were interested in whether dyslexics would show a different entrainment profile at relatively slow temporal rates of amplitude modulation only (<10 Hz), or whether atypical entrainment would be revealed across both slower and faster timescales in speech. Our results were consistent in indicating that Syllable-based timing was most disrupted in developmental dyslexia. Regarding rhythm perception (Experiment 1, tapping), dyslexic participants showed significant entrainment differences for Syllable-rate AM patterns in trochaic sentences, consistently timing their taps to an earlier oscillatory phase compared to the control group. They also showed significant entrainment to trochaic Stress-rate AMs, which control participants did not. Individual differences in preferred Syllable and Stress phase of tapping were related to individual differences in spelling ability and phonological awareness. For rhythmic speech production (Experiment 2), dyslexic participants showed a greater degree of syllable shortening for sentences with greater metrical complexity. Dyslexics also showed an altered phase-locking profile between Syllable- and Phoneme-rate AMs in their utterances. Overall, therefore, the data from these experiments supports a primary impairment in entrainment at slower temporal rates of AM (delta and theta) in developmental dyslexia (Goswami, 2011).

Nevertheless, the potential existence of group differences in phase-locking at the Phoneme rate cannot be ruled out. Firstly, if rhythmic entrainment at *slow* rates is significantly altered in dyslexia, there may be knock-on consequences (i.e. via hierarchical oscillatory nesting) at faster temporal rates as well. Secondly, participants' taps in Experiment 1 at the Phoneme rate were not sufficiently phase-locked for either group to enable further analysis. Therefore, methods in which responses can be measured with greater temporal resolution are needed to investigate Phoneme-rate phase-locking to speech in developmental dyslexia. The absence of group differences in overall entrainment accuracy and entrainment strength should also be noted. A strong entrainment hypothesis (as well as prior behavioural data, e.g. Thomson and Goswami, 2008) would predict group differences in mean intertap-intervals or in cross-frequency synchronisation strength. Further, previous studies have indicated substantial behavioural speech rhythm deficits in dyslexia (Wood and Terrell, 1998; Kitzen, 2001; Goswami et al., 2010, 2013a; Leong et al., 2011a; Holliman et al., 2010, 2012). However, the findings in the current study could reflect our cohort of dyslexic participants, who were all well-compensated adults attending a world-class university. It is possible that differences in entrainment accuracy and strength would be observed for younger participants with dyslexia or for less well-compensated adults with dyslexia.

The earlier Syllable phase of tapping observed in the rhythm perception experiment is consistent with the greater anticipation observed by Wolff (2002) and by Thomson and Goswami (2008) in the tapping responses of adolescent dyslexics. Some anticipation of the beat is typically observed when tapping is the dependent variable. For example, Fraisse (1982) reported that taps produced by neurotypical adults consistently anticipated a metronome signal by around 30 ms. In Thomson and Goswami's (2008) study, dyslexic children aged 10 years anticipated a metronome beat at 2 Hz by 39 ms, compared to 28 ms for control children. Therefore, one explanation for the earlier phase of tapping observed for the dyslexics studied here is that they anticipated the beat more strongly than control participants. However, stronger rhythmic anticipation should also be reflected in stronger phase locking, which was not observed in our data. A second possible explanation is that dyslexics *perceive* the perceptual onset of beats as occurring earlier, as compared to controls. To investigate this possibility, it is necessary to measure P-centre perception in dyslexics using appropriately-designed speech stimuli. A third possibility is that dyslexics might differ in motor control. For example, they may be less able to inhibit latent responses to salient stimuli (stressed syllables). To investigate this motor explanation, one would need to repeat the tapping experiment using electromyography (EMG), in order to examine the timecourse of motor response activation and inhibition directly.

The altered synchronisation *phase* observed for the dyslexic cohort in Experiment 2 is suggestive of differences in their hierarchical organisation or binding of speech sounds. That is, phonological units on different timescales (e.g. phonemes, syllables) may be mis-aligned in dyslexics' phonological representations for spoken words. If speech is concurrently sampled on different timescales (e.g. syllabic and phonemic) within separate neural ‘channels’, these separate streams of information must eventually be bound together in precise temporal alignment to generate the complete speech percept (Poeppel, 2003; Giraud and Poeppel, 2012). Our results suggest that dyslexics may have problems with this temporal binding or alignment of speech sounds, particularly when syllable patterns are involved. For example, a (temporal) phase-shift in Syllable–Phoneme alignment could suggest that consonant onsets are represented neurally as occurring relatively earlier with respect to the overall syllable cycle (particularly with respect to the vowel nucleus).

### 5.2 Links between rhythm perception & production

In this study, we measure differences in auditory rhythmic perception using tasks that require a motor output (e.g. tapping or speaking). Therefore, any differences between controls and dyslexics could either arise from differences in auditory *perception*, or in motor production. For example, according to a motor account, the timing differences observed in dyslexics' speech would not originate from mental differences in phonological representation, but from synchronisation for action of the motor articulators involved in producing syllable- and phoneme-rate speech gestures (e.g. jaw, lips, tongue). Indeed, the accurate production of speech requires precise temporal synchronisation between motor articulators (Kelso et al., 1986), and logically this temporal synchrony could be disrupted in dyslexia. However, since speech perception and production mechanisms are thought to share similar rhythmic constraints and neural representations (Martin, 1972; Liberman and Mattingly, 1985), perceptual and motor accounts of the data are not necessarily mutually exclusive. For example, differences in the neuronal oscillatory architecture of the auditory cortex would be expected to affect both perception (Luo and Poeppel, 2007) and the profile of motor responding (e.g. Henry and Obleser, 2012). However, to rule out a purely low-level motor deficit and, to adjudicate between phonological and motor explanations of the effects observed here, it would be necessary to measure dyslexics' mental phonological representations *directly* without requiring a motor response. Recent advances in methods that use participants' *neural* entrained responses to ‘reconstruct’ the speech envelope (thereby visualising phonological encoding in the brain) provide one means to investigate such representational integrity (Pasley et al., 2012; Ding and Simon, 2012).

### 5.3 Wider implications of a syllable-timing deficit

By hypothesis, the syllable timing differences that we observe here for regular, metronome-timed speech would have important consequences for dyslexics' perception and production of everyday conversational speech, which is not typically metrically- or temporally-regular (Dauer, 1983). In our view, natural speech perception *does* involve use of the rhythmic cues carried by stressed syllables (prosodic structure) to provide a temporal context for speech perception and production, even if these rhythmic cues (i.e. strong syllables) are not perfectly isochronously spaced in time (see Goswami and Leong, 2013). Ours is not an isolated view. One proposed function of prosodic rhythm in speech is to support word segmentation during normal listening (Cutler and Norris, 1988). According to this view, strong syllables trigger segmentation of the speech signal, whereas weak syllables do not. For example, Cutler and Norris (1988) found that the target syllable “mint” was detected more slowly in the nonsense word “mintayve” (s–s) than in the nonsense word “mintesh” (s–w). This outcome was observed because the second strong syllable in “mintayve” triggers an inappropriate segmentation that competes for lexical access with the target. Prosodic stress can also constrain lexical access by selectively activating word candidates with a matching stress template (Cutler, 2005). For example, the syllable “ad” receives primary stress in “admiral”, but secondary stress in “admiration”. Cooper et al. (2002) found that when primed with the fragment “ad” spliced from “admiral” or “admiration”, listeners could make use of the subtle differences in co-articulatory features to activate the appropriate word. Note that both of these rhythm-based effects (word segmentation and cueing for lexical access) are proposed by Cutler and colleagues to occur in everyday speech, and can even operate at the single-word level. Accordingly, if the temporal perception of stressed syllables is altered in dyslexia, this would have cascading effects throughout the phonological hierarchy. The temporal context for representing weak syllables and phonemes would be altered, resulting in altered phonological representations of syllables and words, and word segmentation could be triggered at a different (earlier) point in speech, affecting holistic phonological representations. Note that behavioural studies showing reduced sensitivity to syllable stress in dyslexia have all used natural speech (Goswami et al., 2010, 2013a; Kitzen, 2001; Leong et al., 2011a; Mundy and Carroll, 2012). Importantly, in all these cited studies, dyslexics' poor performance on prosodic tasks was strongly associated with their poor phonological skills.

A syllable timing deficit in dyslexia would also have significant implications for the neural entrainment of oscillatory networks. Our results predict that dyslexics should show altered oscillatory entrainment to rhythmic stimuli, especially regarding theta-rate (syllable-rate) entrainment. Given the wider literature on prosodic deficits in dyslexia, we would predict that altered neuronal entrainment should be observed for natural speech as well as rhythmic speech. This is open to empirical investigation. It would also be interesting to explore the neural substrates underlying the differences in syllable timing observed here. Previous work on beat perception in non-speech sounds has implicated subcortical structures such as the basal ganglia and cerebellum in beat-based and duration-based timing respectively (Grahn, 2009; Teki et al., 2011). Conversely, in the domain of speech processing, attention has recently been focussed on the role of cortical oscillations, whose entrainment to acoustic modulation patterns is thought to support multi-timescale temporal sampling of syllable and phoneme patterns (Poeppel, 2003; Giraud and Poeppel, 2012). In our view, speech perception may be subserved by both cortical and sub-cortical structures (Kotz and Schwartze, 2010). Therefore both types of structures may respond to and use speech rhythm for different computational and functional purposes. Clearly, detailed research is required to identify the particular neural loci that are implicated in the disruptions in syllable- and stress-rate entrainment found in the current study.

In typical language development, children spontaneously acquire syllable awareness and onset-rime awareness in the pre-reading stage (e.g. Treiman and Zukowski, 1991; Ziegler and Goswami, 2005). One proposal is that children's lexical representations become increasingly segmental over development, gradually becoming re-organised from a syllable-based phonology to a segmental phonology (Fowler, 1991). Learning to read is particularly important for the development of phoneme-based representations, with pre-reading children showing little awareness of phonemes (Ziegler and Goswami, 2005). With regard to the temporal modulation structure of speech, these and other developmental data suggest that phonological development may progress hierarchically through the dominant modulation timescales in speech, with phonological representations becoming increasingly mentally elaborated or specified from slow to fast modulation rates. For example, newborn infants are very sensitive to speech rhythm, suggesting early sensitivity to *slow* (∼3–5 Hz) syllable-rate temporal information in speech. These slow AMs may form a crucial part of early phonological representations. If such syllable-based representations were atypical from early in development, this would have developmental consequences throughout the lexical system. Future longitudinal studies incorporating neural measures of oscillatory entrainment could investigate this hypothesis empirically.

### 5.4 Measurement of multi-timescale entrainment using speech AMs

Here we used a novel envelope-based AM method (the S-AMPH model) to examine rhythmic entrainment to multiple timescales in speech. This method (Leong, 2012) complemented more established ways of measuring rhythmic performance, such as the measurement of intervals (e.g. inter-tap intervals) and asynchronies (e.g. distance to P-centres). Across both experiments, the envelope-based AM method provided a robust measure of rhythmic entrainment, revealing group differences that were not identified by conventional methods of analysis. This envelope-based AM method also allowed us to address the theoretical question of whether rhythmic entrainment in dyslexia was impaired on both slow and fast timescales, even when using a complex stimulus like speech. Finally, the envelope-based AM method made use of the *inherent* temporal structure of the speech stimulus to measure entrainment, rather than requiring any additional assumptions to artificially determine the location of target beats (i.e. P-centres). While it is a relatively straightforward process to identify the location of beats in musical stimuli, the equivalent process in speech is much more complex, since the acoustic correlates of P-centres in speech are still unclear (Villing, 2010; Patel et al., 1999).

Our envelope-based AM method expands the repertoire of entrainment indices that can be measured in future rhythm studies. The S-AMPH model provides new indices of rhythmic calibration, such as the strength and angle of phase-locking, and cross-frequency phase synchronisation measures, which were successful in revealing significant patterns in the data. This envelope-based AM method may also be applied to speech that is *not* rhythmically isochronous. For example, the S-AMPH model has previously been used successfully to characterise rhythmic differences between freely-produced child-directed and adult-directed speech (Leong, 2012). In the future, the AM-based methods developed here could be used to identify rhythmic differences in natural and spontaneous utterances by dyslexic individuals.

### 5.5 Potential musical rhythmic intervention strategies for dyslexia

This paper highlights an example of how research into rhythm and entrainment (traditionally investigated in the domain of music) has informed our understanding of language acquisition processes in developmental dyslexia. It also motivates neural hypotheses regarding these rhythmic and entrainment processes, which accordingly can be applied in music research. Indeed, the domain of music may contribute novel rhythm-based intervention strategies in dyslexia which directly impact phonological representations (see Bhide et al., 2013). There is already evidence that rhythm-based training is at least as effective as phonetic-based training in improving dyslexic children's phonological awareness (Thomson et al., 2012). Furthermore, classroom music lessons have been found to be beneficial for dyslexic children's phonological and spelling skills (Overy, 2003). Studies such as these suggest that a combination of musical (i.e. rhythm) and linguistic (i.e. syllabic) approaches could be particularly beneficial for educating children with dyslexia. Theoretically, music-based training would be expected to improve awareness of supra-segmental (i.e. prosody and rhythmic) aspects of language. Combined with additional phonics-based training, which specifically targets the segmental level of phonological representations, music-based interventions that improve syllable timing in dyslexia may offer novel avenues for auditory therapies.

## Figures and Tables

**Fig. A:**
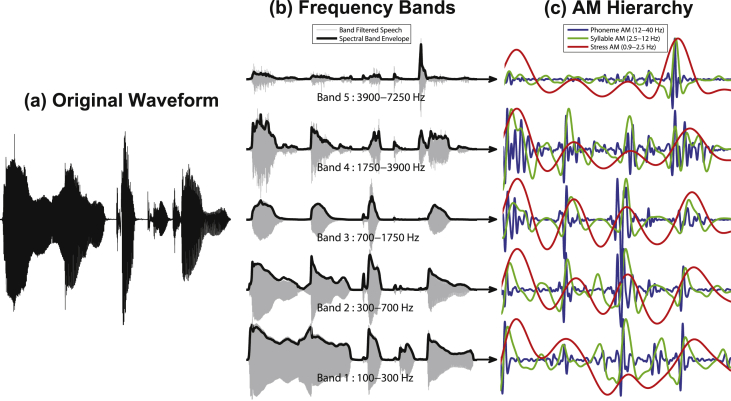
Signal-processing stages in the S-AMPH model. (a) Original acoustic waveform of the spoken sentence “Mary Mary quite contrary”. (b) In the S-AMPH model, the original speech signal is first filtered into 5 frequency bands, and the Hilbert envelope is computed for each frequency band. (c) A 3-tier AM hierarchy is then extracted from the envelopes of each frequency band. The resulting ‘Stress’ (0.9–2.5 Hz), ‘Syllable’ (2.5–12 Hz) and ‘Phoneme’ (12–40 Hz) AMs are shown overlaid in different colours. These correspond to prosodic stress patterns, syllable patterns and phoneme patterns respectively. This results in a 5 (frequency band) × 3 (AM hierarchy) spectro-temporal representation of the speech amplitude envelope.

**Fig. B:**
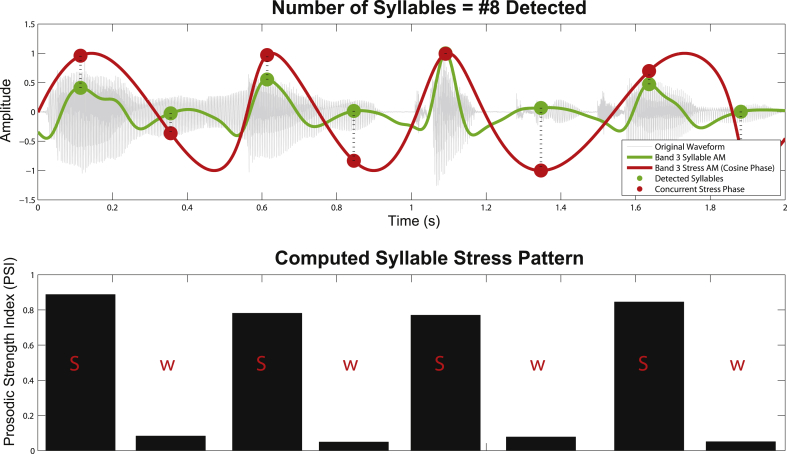
Automatic detection of syllables and prosodic stress pattern for the spoken sentence “Mary Mary quite contrary” using the S-AMPH model. The top panel shows the original waveform (grey), the Syllable AM for spectral band 3 (green line) and the Stress AM cosine phase for spectral band 3 (red line). The 8 peaks in the Syllable AM that are automatically detected (green dots) correspond to the 8 spoken syllables in the sentence. For each Syllable AM peak, the concurrent Stress AM phase is automatically recorded (red dots). These phase values are then used to compute the prosodic strength (0–1) for each syllable. The bottom barchart shows the prosodic strength computed by the S-AMPH model for each of the 8 detected syllables in the utterance. Consistent with the trochaic patterning of the sentence, the computed syllable stress pattern is ‘s–w–s–w–s–w–s–w’.

**Table 1:** Mean tapping phase for each group at each AM rate, for trochaic and iambic sentences.

		Stress AM phase (radians)		Syllable AM phase (radians)		Phoneme AM phase (radians)	
		Controls	Dyslexics	Controls	Dyslexics	Controls	Dyslexics
Trochaic sentences	Mean (SD)	−0.08 π (0.38 π)	−0.08 π (0.33 π)	+0.40 π** (0.28 π)	+0.09 π** (0.31 π)	−0.48 π (0.44 π)	−0.91 π (0.37 π)
Iambic sentences	Mean (SD)	−0.39 π (0.41 π)	−0.73 π (0.36 π)	+0.55 π (0.28 π)	+0.47 π (0.31 π)	+0.75 π (0.43 π)	−0.44 π (0.44 π)

**p < 0.01 for difference between controls and dyslexics.

**Table 2:** Correlation coefficients (*r*-values) for circular–linear correlations between AM phase of tapping, and literacy and phonology measures, for trochaic and iambic sentences. For each variable, correlations were computed in three ways – across all participants (‘All’), across controls only (‘Con’) and across dyslexics only (‘Dys’). Correlation values highlighted in bold achieved p-values under .10.

			Reading	Spelling	Spoonerisms
Stress AM	Trochaic	All	0.17	0.16	0.06
		Con	**0.47**^$^	0.12	0.22
		Dys	0.17	0.23	0.16
	Iambic	All	0.22	0.06	0.39*
		Con	0.38	0.40	0.31
		Dys	0.38	0.11	0.67**
Syllable AM	Trochaic	All	0.15	0.52**	**0.35^**&**^**
		Con	0.25	0.30	0.06
		Dys	0.21	0.42	0.35
	Iambic	All	0.25	0.28	**0.33**^$^
		Con	0.04	0.24	0.14
		Dys	0.38	0.30	0.46
Phoneme AM	Trochaic	All	0.26	0.31	0.32
		Con	0.40	0.07	0.07
		Dys	0.04	0.36	0.44
	Iambic	All	0.17	0.16	0.07
		Con	0.33	0.16	0.12
		Dys	**0.50^**&**^**	0.28	0.11

**p < 0.01. *p < 0.05. ^^**&**^^*p* = 0.07. ^$^*p* = 0.09.

## Appendix 1 The Spectral Amplitude Modulation Phase Hierarchy (S-AMPH) model

For the AM-based multi-timescale analysis, AM patterns on 3 major timescales were extracted from the amplitude envelope of the speech acoustic signal. These AM patterns represent the ‘dominant’ modulation structure in the speech envelope, and form the basis of an amplitude-based model of speech rhythm, the Spectral-Amplitude Modulation Phase Hierarchy (S-AMPH) model (Leong, 2012). In the S-AMPH model, AM patterns at ‘Stress’, ‘Syllable’ and ‘Phoneme’ rates form a 3-tier nested hierarchy, mirroring the linguistic prosodic hierarchy. Oscillatory cycles at each AM rate represent linguistic units of different lengths. Furthermore, phase relationships between the Stress and Syllable AM tiers of the hierarchy are able to specify ‘Strong-weak’ rhythm patterns in a spoken sentence, as described further in Leong (2012) and Goswami and Leong (2013).

Fig. A details the signal-processing stages involved in the S-AMPH model. To obtain the 3-tier AM hierarchy, the raw acoustic signal is first filtered into 5 frequency bands using a series of adjacent finite impulse response (FIR) filters. These 5 frequency bands are: (1) 100–300 Hz; (2) 300–700 Hz; (3) 700–1750 Hz; (4) 1750–3900 Hz; and (5) 3900–7250 Hz. The number of frequency bands and their respective bandwidths were determined in a previous analysis using PCA dimensionality reduction of the envelopes of 29 ERB-spaced frequency channels (spanning 100–7250 Hz). Next, the Hilbert envelope is extracted from each of the 5 sub-band filtered signals. These extracted Hilbert envelopes are then passed through a second series of band-pass filters in order to isolate the 3 different AM rates. These 3 AM rates are the ‘Stress’ rate (0.9–2.5 Hz), ‘Syllable’ rate (2.5–12 Hz) and ‘Phoneme’ rate (12–40 Hz). This second ‘modulation filterbank’ (MFB) also consists of a series of adjacent finite impulse response (FIR) filters. For a detailed description of the MFB design and features, see Stone and Moore (2003, p. 3). The current MFB was adapted from this spectral filterbank for use as a modulation filterbank. As before, the number of modulation bands and their respective bandwidths were determined in a dimensionality-reduction exercise in which 24 finely-spaced modulation channels were reduced to 3 major AM bands. The result of this two-step filtering process is a 5 (frequency band) × 3 (AM hierarchy) spectro-temporal representation of the envelope of the speech signal.

The S-AMPH model takes this 5 × 3 AM spectro-temporal structure as its input and uses this to compute the ‘Strong-weak’ syllable stress patterns present in the stimulus. Briefly, this computation assumes that each prominent peak in the Syllable AM corresponds to a single uttered syllable. The concurrent Stress AM phase at which this Syllable AM peak occurs indexes the prosodic strength of the syllable, on the logic that syllables occurring near a Stress peak (∼0 π rad) will be perceived as loud (strong) while syllables occurring near a Stress trough (−π/π rad) will be perceived as soft (weak). This logic is formalised mathematically using an exponential function, which converts the Stress phase-of-occurrence for each syllable into a ‘prosodic strength index’ ranging from 0 to 1. For example, the computed syllable stress pattern for the trochaic nursery rhyme sentence ‘Mary Mary quite contrary’ is shown in Fig. B, with Stress and Syllable AMs from frequency band 3 only shown as an illustration. In this example, the S-AMPH model extracts the 8 uttered syllables and their alternating ‘s–w’ stress pattern perfectly. Although automatic syllable detection and stress assignment are not the subject of the current study, this exercise demonstrates that the AM patterns used in the current analysis do indeed bear rhythm-relevant information.

For this paper, only AMs from 1 out of the 5 frequency bands were used to computate Stress, Syllable and Phoneme AM phase. This was done to simplify the analysis process. In the rhythm perception experiment (tapping), Spectral Band 3 (700–1750 Hz) was used as this typically contains the strongest vowel energy, and was therefore expected to be the most closely associated with syllable P-centres (perceived beats). In the rhythm production experiment, Spectral Band 1 (100–300 Hz) was used as this was expected to be the most strongly associated with prosodic patterning, consistent with previous experiments using low-pass filtering to isolate prosodic content (e.g. Nazzi et al., 1998).

## Footnotes

1 We also computed these analyses by measuring the intervals between the stressed vowels only. Identical results were obtained, hence we report here the VOI analysis only.
